# Rapidity and Energy Dependencies of Temperatures and Volume Extracted from Identified Charged Hadron Spectra in Proton–Proton Collisions at a Super Proton Synchrotron (SPS)

**DOI:** 10.3390/e25121571

**Published:** 2023-11-22

**Authors:** Pei-Pin Yang, Fu-Hu Liu, Khusniddin K. Olimov

**Affiliations:** 1 Department of Physics, Xinzhou Normal University, Xinzhou 034000, China; peipinyang@xztu.edu.cn; 2 State Key Laboratory of Quantum Optics and Quantum Optics Devices, Institute of Theoretical Physics, Shanxi University, Taiyuan 030006, China; 3 Laboratory of High Energy Physics, Physical-Technical Institute of Uzbekistan Academy of Sciences, Chingiz Aytmatov Str. 2b, Tashkent 100084, Uzbekistan; 4 Department of Natural Sciences, National University of Science and Technology MISIS (NUST MISIS), Almalyk Branch, Almalyk 110105, Uzbekistan

**Keywords:** transverse momentum spectra, identified charged hadrons, effective temperature, kinetic freeze-out temperature, initial temperature, kinetic free-out volume, 12.40.Ee, 13.85.Hd, 24.10.Pa

## Abstract

The standard (Bose–Einstein/Fermi–Dirac, or Maxwell–Boltzmann) distribution from the relativistic ideal gas model is used to study the transverse momentum ($p_{T}$) spectra of identified charged hadrons ($\pi^{-}$, $\pi^{+}$, $K^{-}$, $K^{+}$, $\bar{p}$, and *p*) with different rapidities produced in inelastic proton–proton (pp) collisions at a Super Proton Synchrotron (SPS). The experimental data measured using the NA61/SHINE Collaboration at the center-of-mass (c.m.) energies $\sqrt{s}=6.3$, 7.7, 8.8, 12.3, and 17.3 GeV are fitted well with the distribution. It is shown that the effective temperature ($T_{eff}$ or *T*), kinetic freeze-out temperature ($T_{0}$), and initial temperature ($T_{i}$) decrease with the increase in rapidity and increase with the increase in c.m. energy. The kinetic freeze-out volume (*V*) extracted from the $\pi^{-}$, $\pi^{+}$, $K^{-}$, $K^{+}$, and $\bar{p}$ spectra decreases with the rapidity and increase with the c.m. energy. The opposite tendency of *V*, extracted from the *p* spectra, is observed to be increasing with the rapidity and decreasing with the c.m. energy due to the effect of leading protons.

## 1. Introduction

The existence of confinement and asymptotic freedom in Quantum Chromodynamics (QCD) has led to many conjectures about the thermodynamic and transport properties of hot and dense matter. Because of confinement, nuclear matter should be composed of low-energy hadrons, and it is considered a weakly interacting gas of hadrons. On the other hand, at very high energies, asymptotic freedom means that the interactions between quarks and gluons are very weak, and the nuclear matter is considered as a weakly coupling gas of quarks and gluons. There should be a phase transition between these two configurations, in which the degrees of freedom of hadrons disappear and Quark–Gluon Plasma (QGP) is formed, which is generated at a sufficiently high temperature or density [1,2,3,4,5,6]. QGP existed in the very early universe (a few microseconds after the Big Bang), and some forms of this matter may still exist in the core of neutron stars. Ultra-relativistic heavy-ion collisions have provided opportunities to systematically create and study different phases of bulk nuclear matter.

Several experiments performed at the Super Proton Synchrotron (SPS) [7,8], Relativistic Heavy Ion Collider (RHIC) [2,3,9,10,11,12,13,14,15], and Large Hadron Collider (LHC) [16,17,18,19] have reported abundant experimental data. The system of proton–proton (pp) collisions is usually used as a reference measurement for heavy ion collisions, as it has several valence quarks involved in the collisions. Collective flow is one of the characteristics of the thermal dense medium of this strongly interacting matter. The generated medium expands collectively such that the flow effect is expected to be distinguished from the thermal motion, which reflects the temperature. The heavy ion physics community has been fascinated by observing unexpected collective behavior in high-multiplicity pp collision events. It is therefore necessary and important to study pp collisions.

The transverse momentum ($p_{T}$) spectra of identified charged hadrons produced in relativistic or high-energy collisions contain abundant information on the collision dynamics and the evolution properties of the system from the initial stage to the end of freeze-out phase [20]. Traditionally, it is believed that the flattening of the $p_{T}$ spectra with high multiplicity is a signal for the formation of a mixed phase of de-confined partons and hadrons. In the hydrodynamical model, the slopes of $p_{T}$ spectra are co-determined by the kinetic freeze-out temperature and the transverse expansion flow of the collision system [21]. The study of $p_{T}$ spectra can reveal information related to the effective temperature ($T_{eff}$ or *T*) of the system. A plateau-like region observed in the excitation function of *T* is considered a possible signal for the formation of mixed-phases, similar to the temperature dependence of entropy observed in the first-order phase transition. In addition, in order to understand the phase transition from QGP to hadronic matter, the transverse momentum density is often studied.

In the physical process of high-energy heavy ion collisions, at least four temperatures are often used, namely initial temperature ($T_{i}$), chemical freeze-out temperature ($T_{ch}$), kinetic (or thermal) freeze-out temperature ($T_{0}$), and *T*. These temperatures correspond to different stages of collisions. The excitation degree of the interaction system at the initial stage is described by $T_{i}$, at which hadrons undergo elastic and inelastic interactions in the hadronic medium. Due to the shortage of research methods, there is limited research on $T_{i}$ in the community, which should be based on the $p_{T}$. With the decrease in temperature, the system begins to form hadronic matter and enters the chemical freeze-out stage. Under the condition of maintaining a certain degree of local dynamic equilibrium through quasi-elastic resonance scattering, the final stable hadronic yield has almost no change [22,23,24,25]. The $T_{ch}$, and baryon chemical potential ($\mu_{B}$) at this stage can be obtained by using various thermodynamic models [3,26,27,28]. After the chemical freeze-out stage, the system further expands as the interactions become weak. Finally, the system enters the kinetic freeze-out stage as the elastic collisions between hadrons disappear.

In this paper, the $p_{T}$ spectra of identified charged hadrons ($\pi^{-}$, $\pi^{+}$, $K^{-}$, $K^{+}$, $\bar{p}$, and *p*) with different rapidities produced in inelastic pp collisions at the center-of-mass (c.m.) energies $\sqrt{s}=6.3$, 7.7, 8.8, 12.3, and 17.3 GeV at the SPS [29] are studied, where the c.m. energy is also referred to as collision energy. Although the nonextensive distribution of the Tsallis statistics [30,31,32,33,34,35] has been widely used in recent years, the standard (Bose–Einstein/Fermi–Dirac, or Maxwell–Boltzmann) distribution from the relativistic ideal gas model is still used to extract *T* directly and then to obtain the average transverse momentum ($\langle p_{T}\rangle$), root-mean-square transverse momentum ($\sqrt{\langle p_{T}^{2}\rangle }$), $T_{0}$, and $T_{i}$ indirectly.

The remainder of this paper is structured as follows. The formalism and method are described in Section 2. Results and discussion are provided in Section 3. In Section 4, we summarize our main observations and conclusions.

## 2. Formalism and Method

The particles produced in inelastic pp collisions are thought to be controlled by two main mechanisms or excitation degrees. The low-$p_{T}$ region, which is less than 1–2 GeV/*c* is dominated by the soft excitation process [36,37]. The high-$p_{T}$ region that is more than 1–2 GeV/*c* is governed by the hard scattering process [36,37]. The soft process corresponds to a low excitation degree, and the hard process implies a high excitation degree. The two-mechanism scheme is only one possible choice in understanding particle production. If the particles are distributed in a very wide $p_{T}$ region, one should consider the multiple mechanisms or excitation degrees. If the particles are distributed in a relatively narrow $p_{T}$ region, one may choose the single mechanism or excitation degree. In the two-mechanism scenario, it is currently believed that most light-flavor particles are produced in the soft process. The spectrum in the low-$p_{T}$ region shows exponential behavior, which can be fitted by the thermal distribution [38,39,40]. Heavy-flavor particles and some light-flavor particles are produced in the hard process. The spectrum in high-$p_{T}$ region shows inverse power-law behavior and can be fitted using the Hagedorn [41,42], Tsallis–Levy [31,32], or Tsallis–Pareto-type functions [32,33,34,35].

In this investigation, the light particle spectra in the low-$p_{T}$ region in inelastic pp collisions at the SPS are studied by using the most basic thermal distribution, the standard distribution, which comes from the relativistic ideal gas model. The invariant particle momentum (*p*) distribution described by the standard distribution can be given by [30]

(1) $$\begin{array}{c} E\frac{d^{3}N}{d^{3}p}=\frac{1}{2\pi p_{T}}\frac{d^{2}N}{dydp_{T}}=\frac{gV}{{\left(2\pi \right)}^{3}}E{\left[\exp\left(\frac{E-\mu }{T}\right)+S\right]}^{-1}, \end{array}$$

where *N* is the particle number, *g* is the degeneracy factor, *V* is the volume, μ is the chemical potential,

(2) $$\begin{array}{c} E=\sqrt{p^{2}+m_{0}^{2}}=m_{T}\cosh y \end{array}$$

is the energy,

(3) $$\begin{array}{c} m_{T}=\sqrt{p_{T}^{2}+m_{0}^{2}} \end{array}$$

is the transverse mass,

(4) $$\begin{array}{c} y=\frac{1}{2}\ln\left(\frac{1+\beta_{z}}{1-\beta_{z}}\right)=\tanh^{-1}\left(\beta_{z}\right) \end{array}$$

is the rapidity, $\beta_{z}$ is the longitudinal velocity, and S=−1, 1, and 0 correspond to the Bose–Einstein, Fermi–Dirac, and Maxwell–Boltzmann statistics, respectively.

For the wide $p_{T}$ spectra, if a multi-component standard distribution

(5) $$\begin{array}{cc} E\frac{d^{3}N}{d^{3}p}= & \frac{1}{2\pi p_{T}}\frac{d^{2}N}{dydp_{T}}\\ = & \sum_{i=1}^{n}\frac{gV_{i}}{{\left(2\pi \right)}^{3}}E{\left[\exp\left(\frac{E-\mu }{T_{i}}\right)+S\right]}^{-1} \end{array}$$

can be used in the fit, one may obtain multiple temperatures, that is, the temperature fluctuation. Here, *n* denotes the number of components. Let $k_{i}$ (i=1, 2, …, *n*) denote the relative fraction of the *i*-th component, and $V_{i}$ and $T_{i}$ are the volume and temperature corresponding to the *i*-th component, respectively. Naturally, one has

(6) $$\begin{array}{c} V=\sum_{i=1}^{n}V_{i},\;\;T=\sum_{i=1}^{n}k_{i}T_{i},\;\;\sum_{i=1}^{n}k_{i}=1. \end{array}$$

Here, $k_{i}=V_{i}/V$.

Because of the temperature fluctuation, there are interactions among different subsystems or local sources due to the exchange of heat energy. This causes the couplings of entropy functions of various subsystems. The total entropy is then the sum of the entropies of subsystems plus the entropies of the couplings. The temperature fluctuation in the multi-component standard distribution is a way to explain the origin of the Tsallis distribution. Generally, the $p_{T}$ spectra, which can be fitted using the multi-component standard distribution, can also be fitted using the Tsallis distribution. Because of the influence of the entropy index (*q*), the temperature value extracted from the Tsallis distribution is smaller than that from the multi-component standard distribution. In fact, in the fit using the Tsallis distribution, increasing *T* and/or *q* can increase the particle yield in the high-$p_{T}$ region conveniently.

The data sample analyzed in the present work is in the low-$p_{T}$ region. This implies that the standard distribution can be used. In the standard distribution, the unit-density function of *y* and $p_{T}$ is written as

(7) $$\begin{array}{cc} \frac{d^{2}N}{dydp_{T}}= & \frac{gV}{{\left(2\pi \right)}^{2}}p_{T}m_{T}\cosh y\\ & \times {\left[\exp\left(\frac{m_{T}\cosh y-\mu }{T}\right)+S\right]}^{-1}. \end{array}$$

Then, the density function of $p_{T}$ is

(8) $$\begin{array}{cc} \frac{dN}{dp_{T}}= & \frac{gV}{{\left(2\pi \right)}^{2}}p_{T}m_{T}\int_{y_{\min}}^{y_{\max}}\cosh y\\ & \times {\left[\exp\left(\frac{m_{T}\cosh y-\mu }{T}\right)+S\right]}^{-1}dy, \end{array}$$

where $y_{\min}$ and $y_{\max}$ are the minimum and maximum rapidities in the rapidity interval, respectively. The density function of *y* is

(9) $$\begin{array}{cc} \frac{dN}{dy}= & \frac{gV}{{\left(2\pi \right)}^{2}}\cosh y\int_{0}^{p_{T\max}}p_{T}m_{T}\\ & \times {\left[\exp\left(\frac{m_{T}\cosh y-\mu }{T}\right)+S\right]}^{-1}dp_{T}, \end{array}$$

where $p_{T\max}$ is the maximum $p_{T}$ in the considered rapidity interval. Although $p_{T\max}$ can be mathematically infinite, it is only large enough in physics due to the limitations of the conservation of energy and momentum.

No matter what the specific form of particle momentum distribution is used, the probability density function of $p_{T}$ is written in general as

(10) $$\begin{array}{cc} f(p_{T})= & \frac{1}{N}\frac{dN}{dp_{T}}. \end{array}$$

Naturally, $f(p_{T})$ is normalized to 1. That is,

(11) $$\begin{array}{c} \int_{0}^{\infty }f\left(p_{T}\right)dp_{T}=1. \end{array}$$

One has the average transverse momentum,

(12) $$\begin{array}{c} \langle p_{T}\rangle =\frac{\int_{0}^{\infty }p_{T}f\left(p_{T}\right)dp_{T}}{\int_{0}^{\infty }f\left(p_{T}\right)dp_{T}}=\int_{0}^{\infty }p_{T}f\left(p_{T}\right)dp_{T}, \end{array}$$

and the root-mean-square $p_{T}$,

(13) $$\begin{array}{c} \sqrt{\langle p_{T}^{2}\rangle }=\sqrt{\frac{\int_{0}^{\infty }p_{T}^{2}f\left(p_{T}\right)dp_{T}}{\int_{0}^{\infty }f\left(p_{T}\right)dp_{T}}}=\sqrt{\int_{0}^{\infty }p_{T}^{2}f\left(p_{T}\right)dp_{T}}. \end{array}$$

In principle, there are three independent chemical potentials, baryon ($\mu_{B}$), electric charge or isospin ($\mu_{I}$), and strangeness ($\mu_{S}$), which are related to the three conserved charges. Although the chemical potential, $\mu_{\pi }$ ($\mu_{K}$ or $\mu_{p}$), of the pion (kaon or proton) can be written in terms of the above three chemical potentials [43,44,45,46,47,48,49], we obtained them by using an alternative method in the present work for more convenience.

Considering the yield ratio [$k_{j}$ (j=π, *K*, and *p*)] of negatively to positively charged hadrons ($j^{-}$ to $j^{+}$), the corresponding chemical potentials ($\mu_{j^{-}}$ and $\mu_{j^{+}}$), and the corresponding source temperature ($T_{j^{-}}$ and $T_{j^{+}}$), one has that the relationship between $k_{j}$ and $\mu_{j}$ is [20,50,51,52,53]

(14) $$\begin{array}{c} k_{j}\equiv \frac{j^{-}}{j^{+}}=\exp\left(\frac{\mu_{j^{-}}}{T_{j^{-}}}-\frac{\mu_{j^{+}}}{T_{j^{+}}}\right)=\exp\left(-\frac{2\mu_{j}}{T_{j}}\right) \end{array}$$

if the conditions

(15) $$\begin{array}{c} T_{j^{-}}=T_{j^{+}}=T_{j},\;\;\mu_{j^{-}}=-\mu_{j^{+}}=-\mu_{j} \end{array}$$

are satisfied. Here, $j^{-}$ and $j^{+}$ also denote the yields of negative and positive hadrons respectively. $k_{j}$ can be obtained simply from the experimental data, and $T_{j}$ should be the chemical kinetic-freezing temperature $T_{ch}$, which is slightly larger than or equal to the effective temperature *T* due to the short lifetime of the system formed in pp collisions. One has $T_{j}\approx T$ in this work.

Further, one has

(16) $$\begin{array}{c} \mu_{j}=-\frac{1}{2}T_{j}\ln k_{j}. \end{array}$$

Obviously, $\mu_{j}$ is energy-dependent due to $T_{j}$ and $k_{j}$ being energy-dependent. Based on a collection of large amounts of experimental data, our previous work [52,53] presents the excitation functions of $\mu_{j}$ in pp and central heavy ion collisions, which can be used for a direct extraction for this study. In particular, $\mu_{j}$ decreases quickly with the increase in energy in pp collisions in the concerned SPS energy range. However, the tendency of $\mu_{\pi }$ in central heavy ion collisions is opposite to that in pp collisions, though the tendency of $\mu_{K}$ is similar, and that of $\mu_{p}$ is also similar in the two collisions. The three $\mu_{j}$ in both the collisions are close to 0 at around 100 GeV and above.

The chemical freeze-out temperature $T_{ch}$ in central heavy ion collisions is also energy-dependent [43,44,45,46,47,48,49], which shows a tendency for a rapid increase at a few GeV and then saturation at dozens of GeV and above. In view of the fact that the tendency of $T_{ch}$ has a parameterized excitation function with unanimity in the community, the present work does not study $T_{ch}$ parameter.

Generally, the kinetic freeze-out temperature $T_{0}$ has a tendency of a rapid increase at a few GeV, and then an ambiguous tendency (increase, decrease, or saturation) appears at dozens of GeV and above. It is worth studying the tendency of $T_{0}$ further. A thermal-related method shows that [54]

(17) $$\begin{array}{c} T_{0}=\frac{\langle p_{T}\rangle }{2\kappa_{0}}, \end{array}$$

where $\kappa_{0}=3.07$ is a coefficient, and a value 2 is introduced by us because two participant partons (one from the projectile and the other from the target) are assumed to contribute to $\langle p_{T}\rangle$. This formula gives an approximate consistent tendency of $T_{0}$ as another thermal-related method [55], which shows $T_{0}$ to be proportional to $\langle p_{T}\rangle$ and the coefficient to be energy-related, though the results from the two methods are not the same.

The initial temperature $T_{i}$, which is comparable to the experimental data, is less studied in the community. According to the string percolation model [56,57,58], $T_{i}$ is expressed as

(18) $$\begin{array}{c} T_{i}=\sqrt{\frac{\langle p_{T}^{2}\rangle }{2F(\xi )}}, \end{array}$$

where

(19) $$\begin{array}{c} F\left(\xi \right)=\sqrt{\frac{1-\exp(-\xi )}{\xi }} \end{array}$$

is the color-suppression factor related to the dimensionless percolation density parameter ξ. In pp collisions, F(ξ)∼1 due to the low string overlap probability. As an initial quantity, $T_{i}$ should reflect the excitation degree of the system at the parton level. Correspondingly, the final quantity $T_{0}$ should also be extracted at the parton level. This is also the reason that the value of 2 is introduced by us in the denominator of the $T_{0}$ expression if one assumes that two participant partons are the energy sources in the formation of a particle.

The kinetic energy of a particle’s directional movement should not be reflected in the temperature parameters. The experimental data used in this paper were all measured in the forward-rapidity region. In order to remove the influence of directional motion, one can directly shift the forward rapidity and its interval to the mid-rapidity with the same interval width during the fitting process. In this paper, we integrate *y* from $y_{\min}=-0.1$ to $y_{\max}=0.1$ in the fit to give a more accurate result, though y≈0 and coshy≈1 near the mid-rapidity. The small difference (<1%) between the accurate and approximate calculations appears mainly in the normalization but not in the temperature parameter.

The method of least squares based on obtaining the minimum $\chi^{2}$ is adopted to obtain the best parameters and their uncertainties. The treatment method is given in Appendix A.

## 3. Results and Discussion

**Figure 1:**
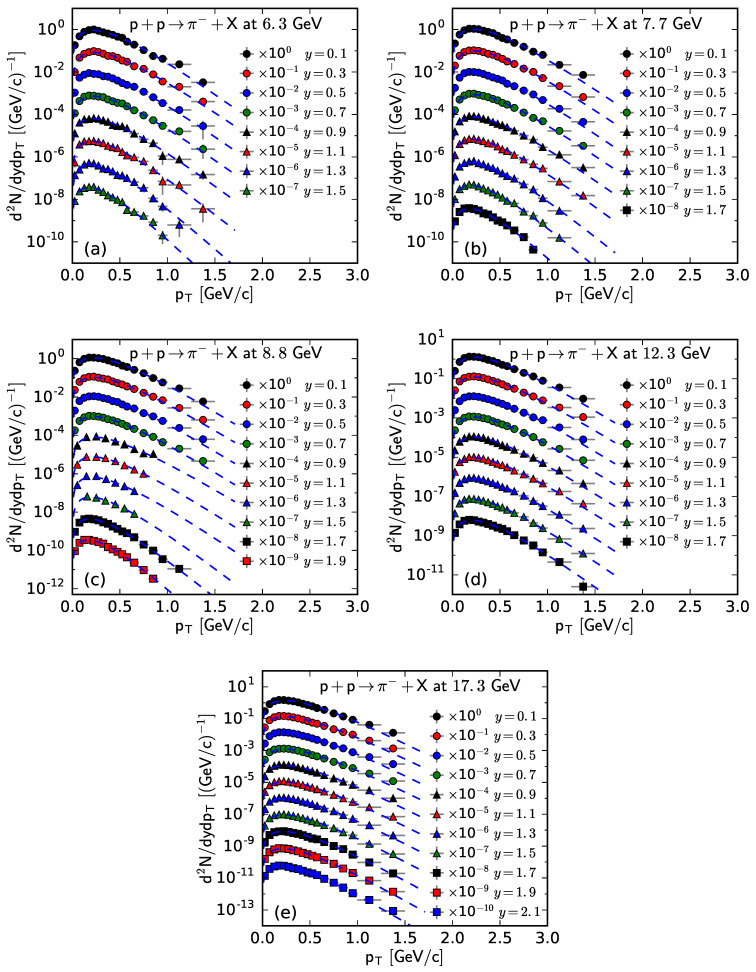
The spectra of $\pi^{-}$ produced in pp collisions at $\sqrt{s}=$ (**a**) 6.3, (**b**) 7.7, (**c**) 8.8, (**d**) 12.3, and (**e**) 17.3 GeV at different *y* with an interval width of 0.2. The symbols represent the experimental data measured by the NA61/SHINE Collaboration [29] and the curves are the fitting results from the Bose–Einstein distribution.

**Figure 2:**
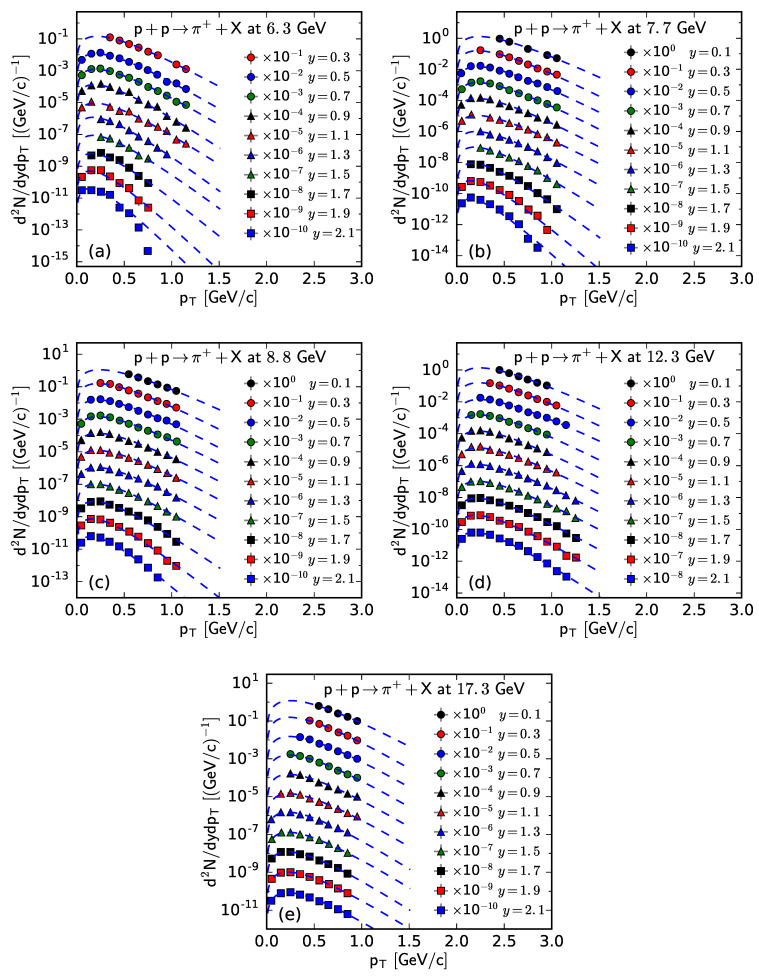
The spectra of $\pi^{+}$ produced in pp collisions at $\sqrt{s}=$ (**a**) 6.3, (**b**) 7.7, (**c**) 8.8, (**d**) 12.3, and (**e**) 17.3 GeV at different *y*. The symbols represent the experimental data measured by the NA61/SHINE Collaboration [29] and the curves are the fitting results from the Bose–Einstein distribution.

Figure 1 and Figure 2 show the rapidity-dependent double differential $p_{T}$ spectra, $d^{2}N/dydp_{T}$, of $\pi^{-}$ and $\pi^{+}$ respectively, produced in inelastic pp collisions at the SPS. Panels (a)–(e) correspond to the results of $\sqrt{s}=6.3$, 7.7, 8.8, 12.3, and 17.3 GeV, respectively. The symbols represent the experimental data at different *y*, with an interval width of 0.2 units, measured using the NA61/SHINE Collaboration [29], and the curves are our results fitted from the Bose–Einstein distribution. In order to see the fitting effect more clearly, the experimental data and fitting results at different rapidities are multiplied by different factors labeled in the panel for scaling. The values of related free parameters (*T*), the normalization constant (*V*), $\chi^{2}$, and the number of degrees of freedom (ndof) for the curves in Figure 1 and Figure 2 are listed in Table A1 in Appendix B. One can see that the fitting results with the Bose–Einstein distribution are in good agreement with the experimental data of $\pi^{-}$ and $\pi^{+}$ spectra, measured using the NA61/SHINE Collaboration in pp collisions at different $\sqrt{s}$ and in different *y* intervals.

**Figure 3:**
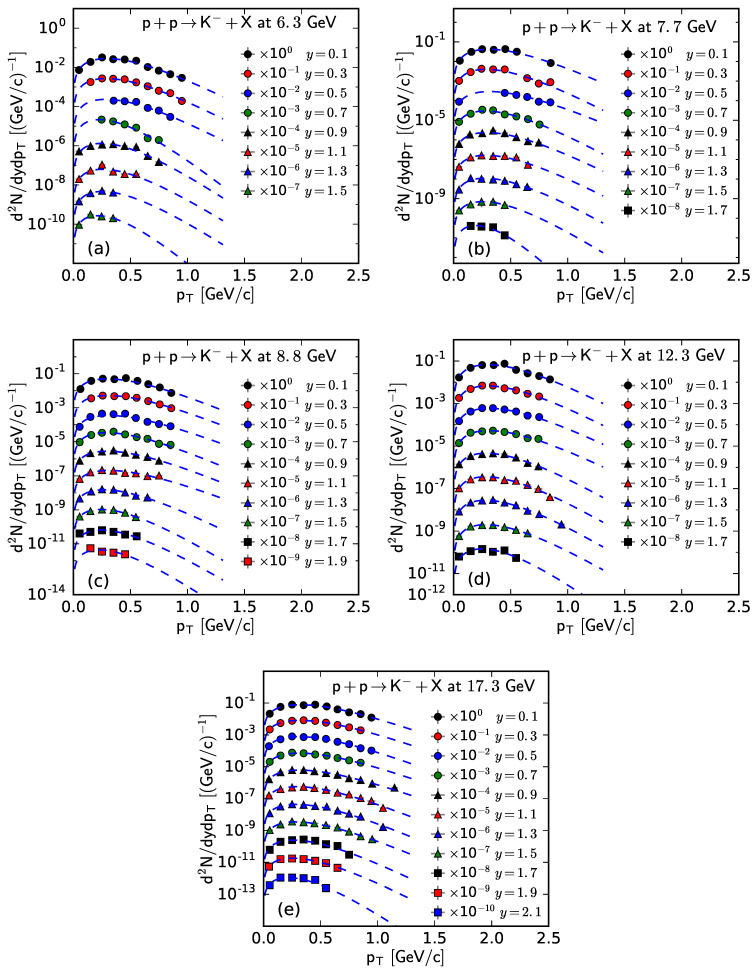
The spectra of $K^{-}$ produced in pp collisions at $\sqrt{s}=$ (**a**) 6.3, (**b**) 7.7, (**c**) 8.8, (**d**) 12.3, and (**e**) 17.3 GeV at different *y*. The symbols represent the experimental data measured by the NA61/SHINE Collaboration [29] and the curves are the fitting results from the Bose–Einstein distribution.

**Figure 4:**
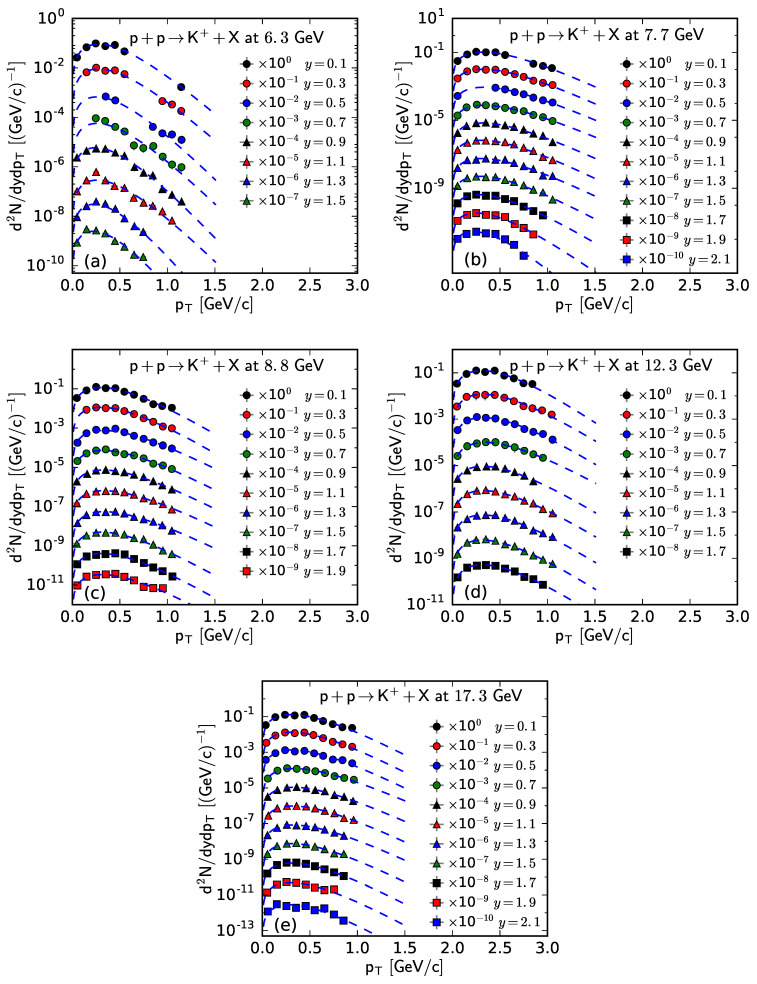
The spectra of $K^{+}$ produced in pp collisions at $\sqrt{s}=$ (**a**) 6.3, (**b**) 7.7, (**c**) 8.8, (**d**) 12.3, and (**e**) 17.3 GeV at different *y*. The symbols represent the experimental data measured by the NA61/SHINE Collaboration [29] and the curves are the fitting results from the Bose–Einstein distribution.

Similarly, Figure 1, Figure 2, Figure 3 and Figure 4 show the rapidity-dependent $d^{2}N/dydp_{T}$ of $K^{-}$ and $K^{+}$, respectively, produced in inelastic pp collisions at different $\sqrt{s}$. The values of *T*, *V*, and $\chi^{2}$/ndof for the curves in Figure 3 and Figure 4 are listed in Table A2 in Appendix B. One can see that the fitting results from the Bose–Einstein distribution are in agreement with the experimental data of $K^{-}$ and $K^{+}$, measured by the NA61/SHINE Collaboration in pp collisions at different $\sqrt{s}$ and in different *y* intervals.

**Figure 5:**
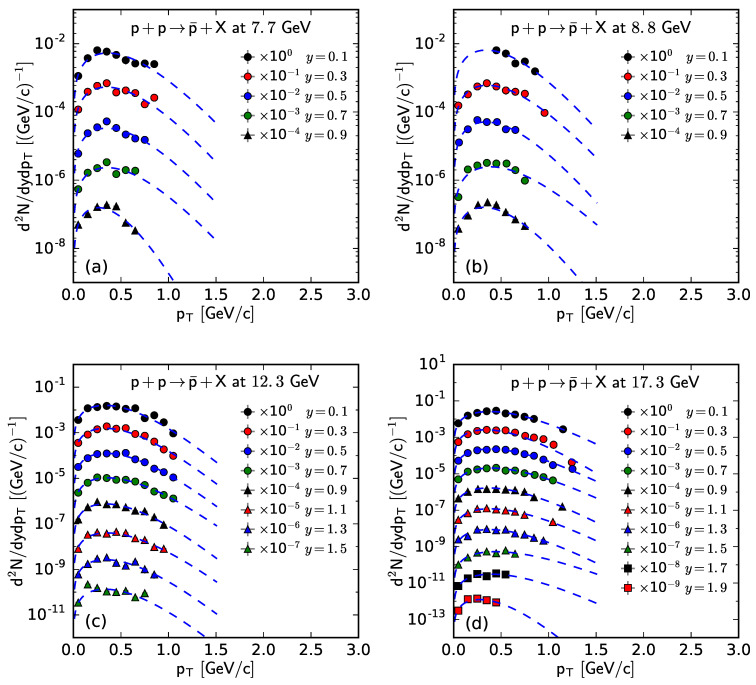
The spectra of $\bar{p}$ produced in pp collisions at $\sqrt{s}=$ (**a**) 7.7, (**b**) 8.8, (**c**) 12.3, and (**d**) 17.3 at different *y*. The symbols represent the experimental data measured by the NA61/SHINE Collaboration [29] and the curves are the fitting results from the Fermi–Dirac distribution.

**Figure 6:**
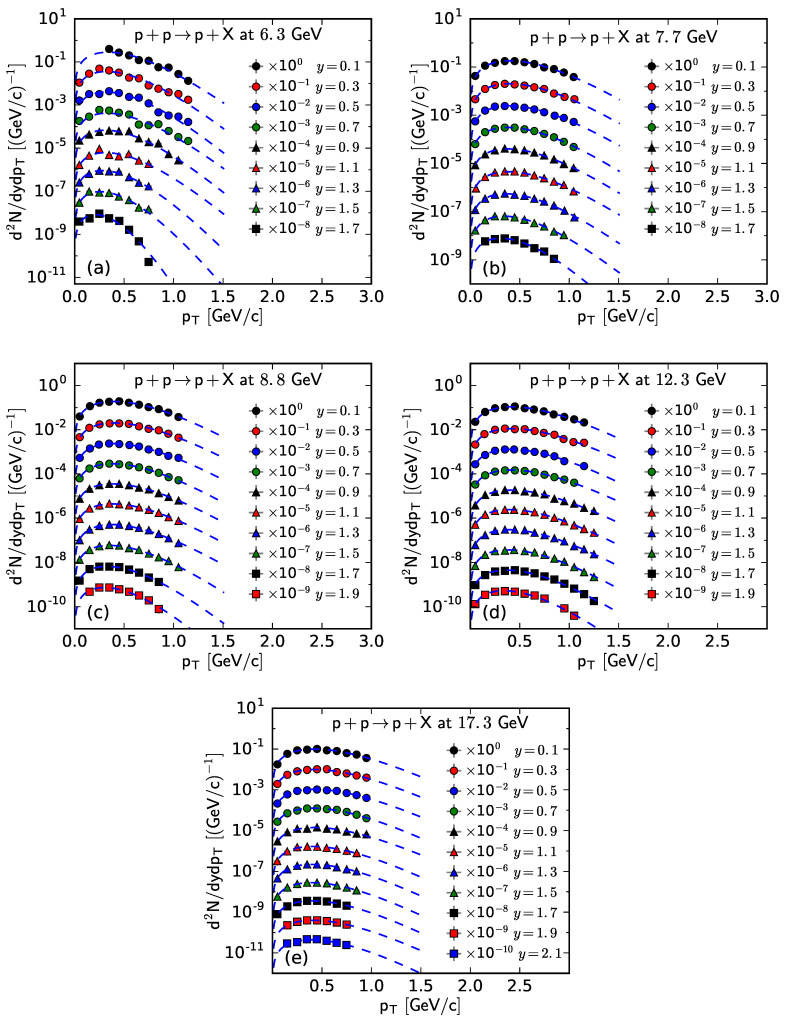
The spectra of *p* produced in pp collisions at $\sqrt{s}=$ (**a**) 6.3, (**b**) 7.7, (**c**) 8.8, (**d**) 12.3, and (**e**) 17.3 GeV at different *y*. The symbols represent the experimental data measured by the NA61/SHINE Collaboration [29] and the curves are the fitting results from the Fermi–Dirac distribution.

Similar to Figure 1, Figure 2, Figure 3 and Figure 4, Figure 5 and Figure 6 show the rapidity-dependent $d^{2}N/dydp_{T}$ of $\bar{p}$ and *p*, respectively, produced in inelastic pp collisions at different $\sqrt{s}$. The experimental data of $\bar{p}$ at $\sqrt{s}=6.3$ GeV in Figure 5 are not available. The values of *T*, *V*, and $\chi^{2}$/ndof for the curves in Figure 5 and Figure 6 are listed in Table A3 in Appendix B. One can see that the $p_{T}$ spectra of $\bar{p}$ and *p* in pp collisions are shown to obey approximately the Fermi–Dirac distribution.

**Figure 7:**
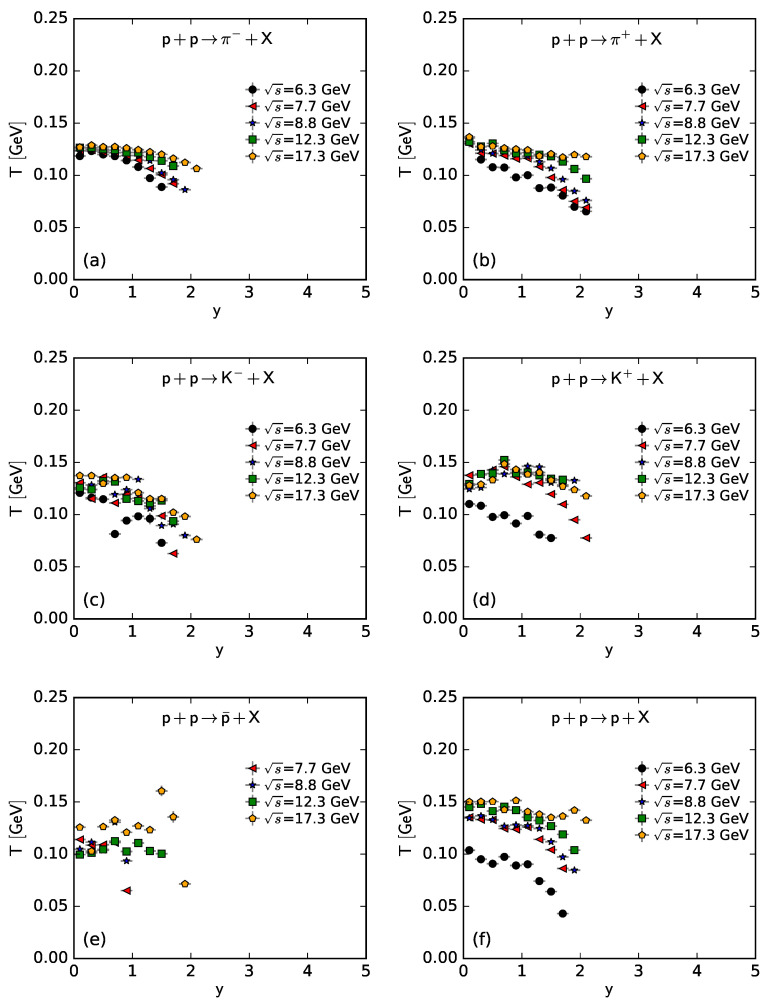
Dependence of *T* on *y* at different $\sqrt{s}$ from the spectra of (**a**) $\pi^{-}$, (**b**) $\pi^{+}$, (**c**) $K^{-}$, (**d**) $K^{+}$, (**e**) $\bar{p}$, and (**f**) *p*.

**Figure 8:**
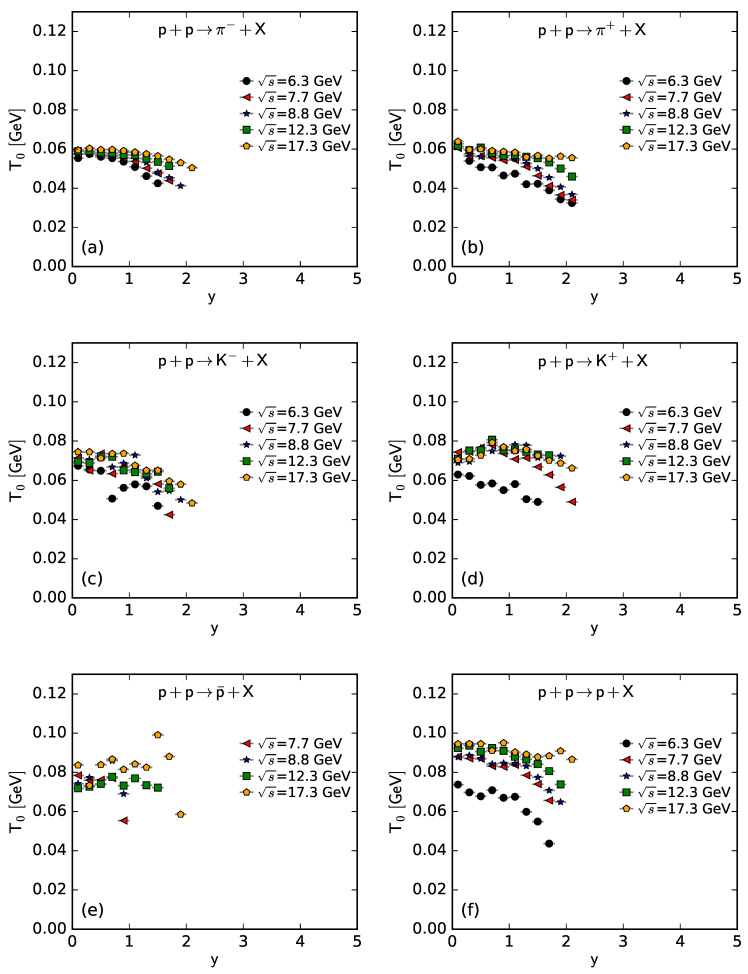
Dependence of $T_{0}$ on *y* at different $\sqrt{s}$ from the spectra of (**a**) $\pi^{-}$, (**b**) $\pi^{+}$, (**c**) $K^{-}$, (**d**) $K^{+}$, (**e**) $\bar{p}$, and (**f**) *p*.

**Figure 9:**
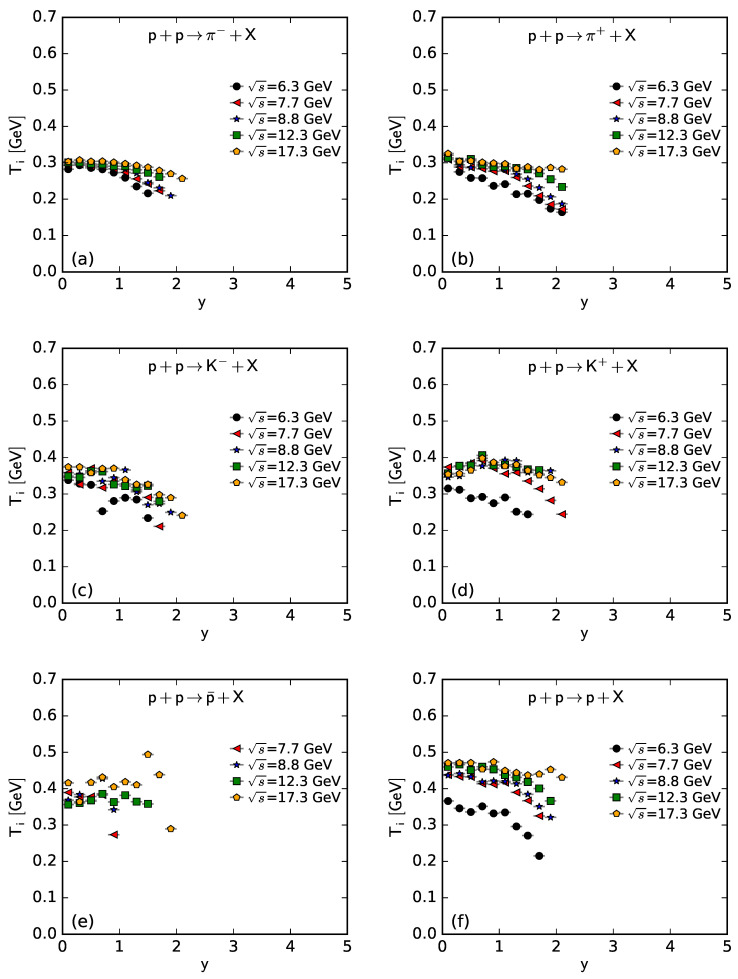
Dependence of $T_{i}$ on *y* at different $\sqrt{s}$ from the spectra of (**a**) $\pi^{-}$, (**b**) $\pi^{+}$, (**c**) $K^{-}$, (**d**) $K^{+}$, (**e**) $\bar{p}$, and (**f**) *p*.

**Figure 10:**
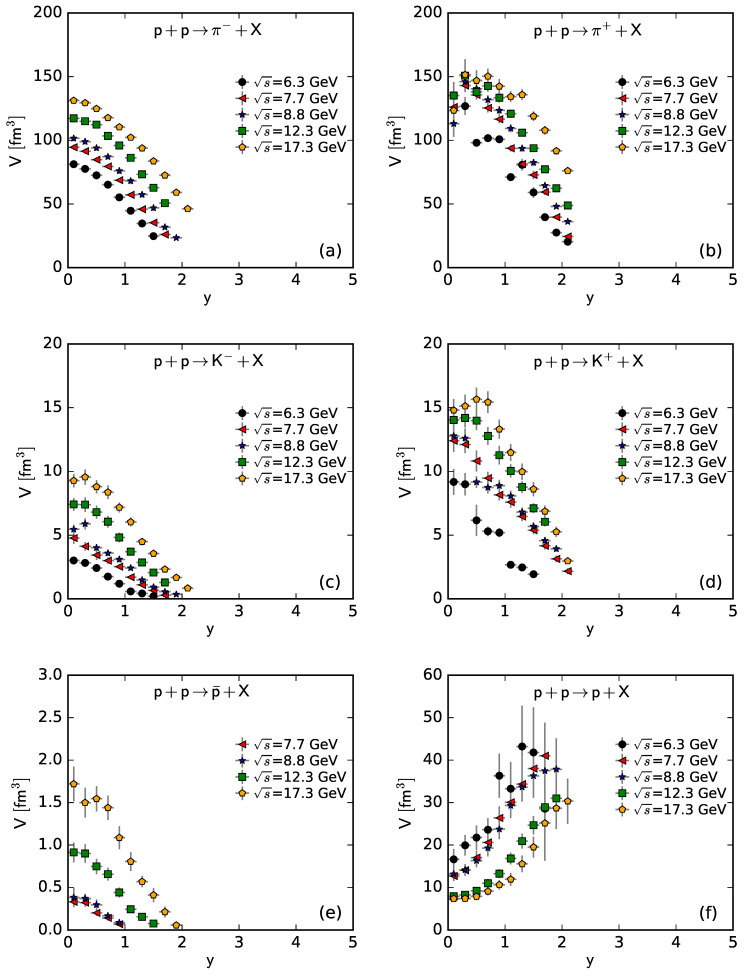
Dependence of *V* on *y* at different $\sqrt{s}$ from the spectra of (**a**) $\pi^{-}$, (**b**) $\pi^{+}$, (**c**) $K^{-}$, (**d**) $K^{+}$, (**e**) $\bar{p}$, and (**f**) *p*.

**Figure 11:**
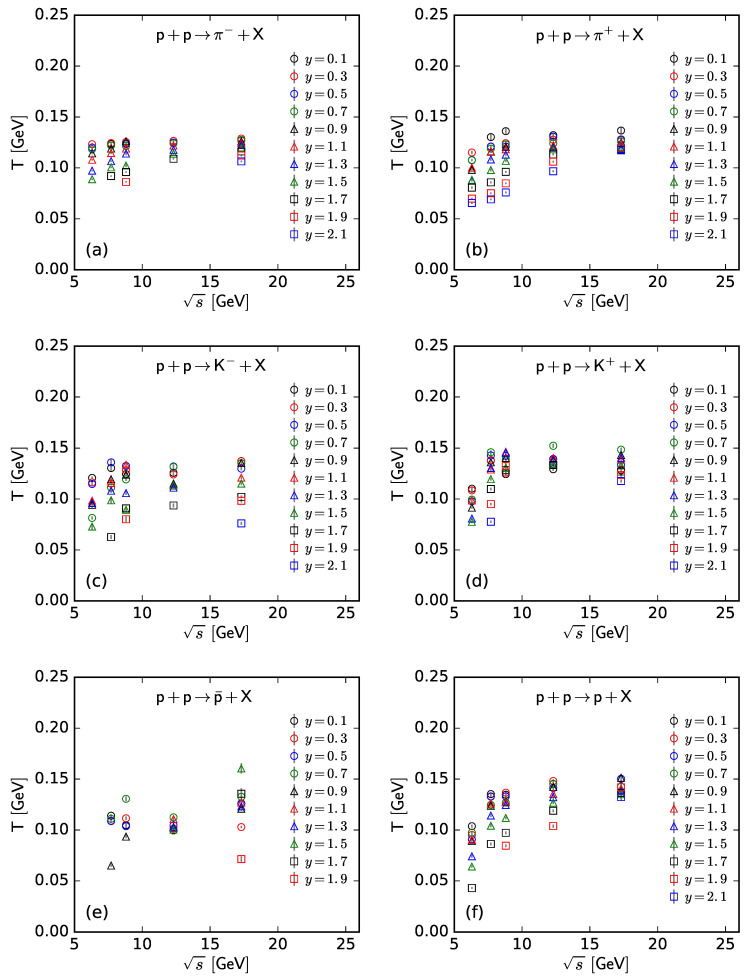
Dependence of *T* on $\sqrt{s}$ at different *y* from the spectra of (**a**) $\pi^{-}$, (**b**) $\pi^{+}$, (**c**) $K^{-}$, (**d**) $K^{+}$, (**e**) $\bar{p}$, and (**f**) *p*.

**Figure 12:**
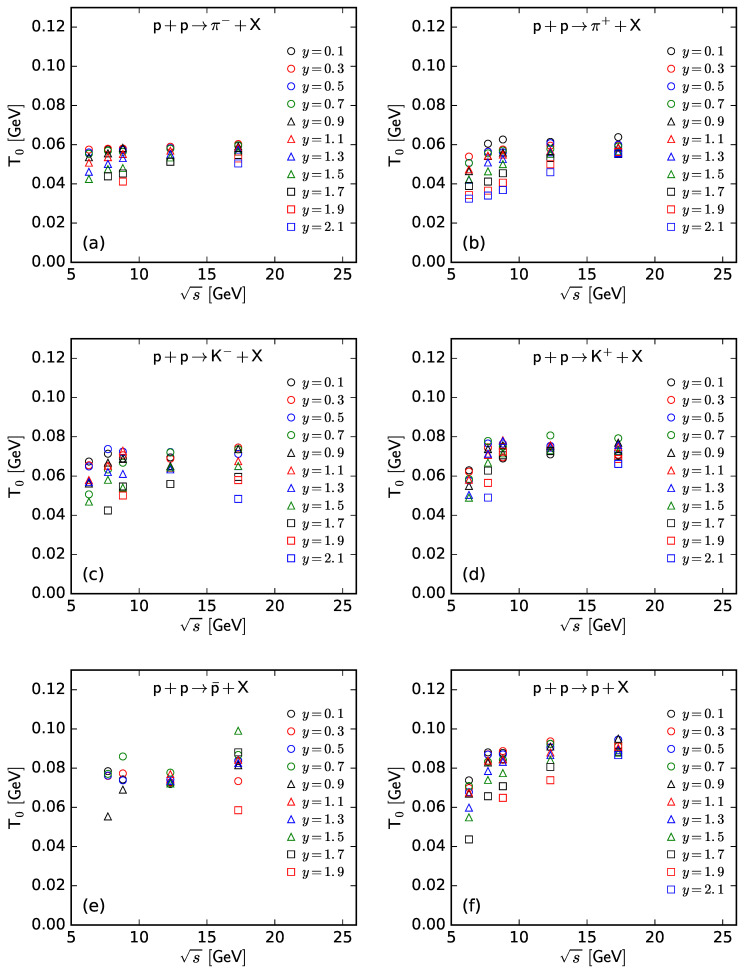
Dependence of $T_{0}$ on $\sqrt{s}$ at different *y* from the spectra of (**a**) $\pi^{-}$, (**b**) $\pi^{+}$, (**c**) $K^{-}$, (**d**) $K^{+}$, (**e**) $\bar{p}$, and (**f**) *p*.

**Figure 13:**
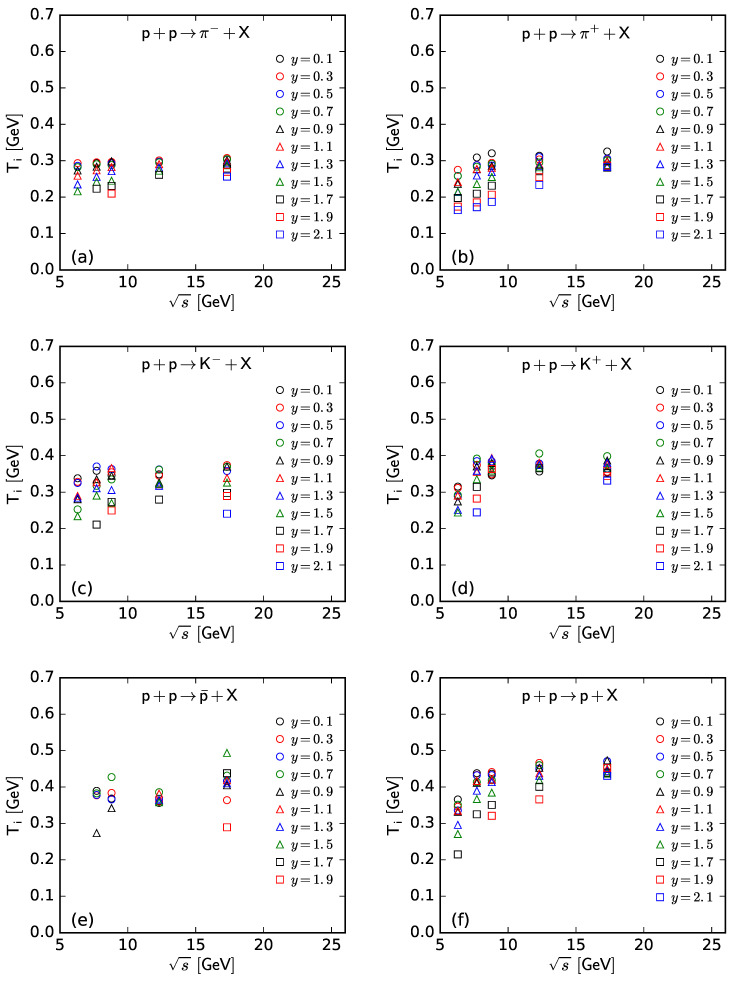
Dependence of $T_{i}$ on $\sqrt{s}$ at different *y* from the spectra of (**a**) $\pi^{-}$, (**b**) $\pi^{+}$, (**c**) $K^{-}$, (**d**) $K^{+}$, (**e**) $\bar{p}$, and (**f**) *p*.

**Figure 14:**
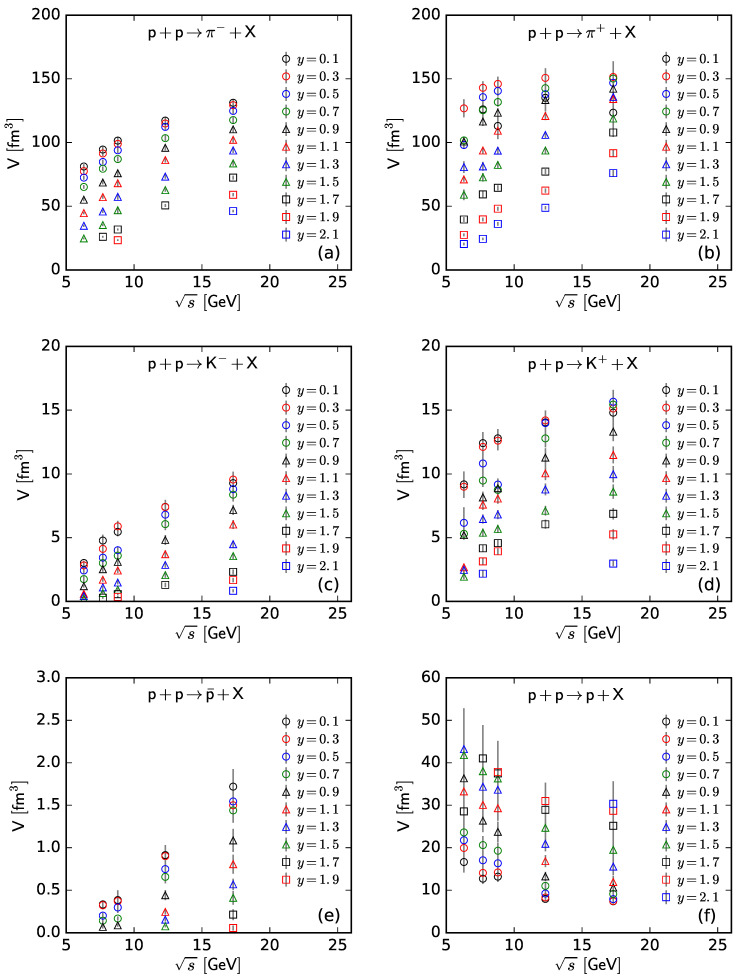
Dependence of *V* on $\sqrt{s}$ at different *y* from the spectra of (**a**) $\pi^{-}$, (**b**) $\pi^{+}$, (**c**) $K^{-}$, (**d**) $K^{+}$, (**e**) $\bar{p}$, and (**f**) *p*.

To show more intuitively the dependence of the free parameter *T* and derived quantities (the kinetic freeze-out temperature $T_{0}$ and initial temperature $T_{i}$) on rapidity, *y*, and c.m. energy, $\sqrt{s}$, Figure 7, Figure 8, Figure 9 and Figure 10 show the relations of *T*–*y*, $T_{0}$–*y*, $T_{i}$–*y*, and *V*–*y* at different $\sqrt{s}$, respectively, and Figure 11, Figure 12, Figure 13 and Figure 14 show the relations of *T*–$\sqrt{s}$, $T_{0}$–$\sqrt{s}$, $T_{i}$–$\sqrt{s}$, and *V*–$\sqrt{s}$ at different *y*, respectively. Panels (a)–(f) correspond to the results from $\pi^{-}$, $\pi^{+}$, $K^{-}$, $K^{+}$, $\bar{p}$, and *p* spectra, respectively. These figures show some changing trends of parameters.

In most cases, one can generally see that *T*, $T_{0}$, and $T_{i}$ decrease (increase) with the increase in *y* ($\sqrt{s}$). There is a tendency of saturation for the three temperatures at $\sqrt{s}=7.7$ GeV and above. Being the initial energy of a saturation effect, 7.7 GeV is a special energy at which the reaction products are proton-dominated and above which the products are meson-dominated. For $\pi^{-}$, $\pi^{+}$, $K^{-}$, $K^{+}$, and $\bar{p}$ spectra, the extracted *V* also decreases (increases) with the increase in *y* ($\sqrt{s}$). However, for *p* spectra, the extracted *V* shows an opposite tendency, increasing (decreasing) with the increase in *y* ($\sqrt{s}$).

There is an isospin and mass independence of *T*. This property is exactly that of $T_{ch}$, which implies a single scenario of chemical freeze-out. However, although $T_{0}$ and $T_{i}$ are isospin-independent, they increase with the increase in mass. The mass dependence of $T_{0}$ is a reflection of a mass-dependent differential kinetic freeze-out scenario or multiple kinetic freeze-out scenarios. The mass dependence of $T_{i}$ means that the formation moments of different particles are different. With the increase in $T_{0}$ ($T_{i}$), massive particles are emitted (formed) earlier. On average, this work shows that $\bar{p}\left(p\right)$ are emitted (formed) earlier than $K^{\mp }$, and $K^{\mp }$ are emitted (formed) earlier than $\pi^{\mp }$, though the relaxation times for the emissions (formations) of different particles can overlap.

Except for *V* from the *p* spectra, the tendencies of other parameters from the *p* spectra, and the tendencies of parameters from the spectra of other particles are easy to understand. It is expected that the local system in the mid-rapidity region has more deposited energy than that in the forward region. Meanwhile, the collision system at a higher energy has more deposited energy than that at lower energy. This results in a higher excitation degree (then higher temperature) at the mid-rapidity and more produced particles (then larger volume) at a higher energy.

The *V* tendency from the *p* spectra is opposite to that from the spectra of other particles. The reason is that the pre-existing leading protons affect the *p* spectra. Because of the leading protons appearing in the forward region, the number of protons and then the volume of a proton source in the fixed interval are small at the mid-rapidity. At a higher energy, the leading protons appear in the more forward region, which leads to a smaller *V* in the fixed interval in the rapidity space. In the present work, the fixed interval is that $\Delta y=y_{\max}-y_{\min}=0.2$.

The values of *V* depend on particle mass and charge. Excluding the case of *p*, which contains pre-existing leading protons in the pp system, *V* decreases significantly with the increase in mass, and positive hadrons correspond to the larger *V* of the emission source. This is because the larger the mass, the more difficult it is to produce this particle. Meanwhile, there is an electromagnetic exclusion (attraction) between positive (negative) hadrons and pre-existing protons. This causes larger (smaller) *V* with an emission source of positive (negative) hadrons.

Generally, the effective temperature *T* is proportional to the mean transverse momentum $\langle p_{T}\rangle$. The present work shows that $T_{\pi^{-}}\approx 0.351{\langle p_{T}\rangle }_{\pi^{-}}$, $T_{\pi^{+}}\approx 0.348{\langle p_{T}\rangle }_{\pi^{+}}$, $T_{K^{-}}\approx 0.284{\langle p_{T}\rangle }_{K^{-}}$, $T_{K^{+}}\approx 0.293{\langle p_{T}\rangle }_{K^{+}}$, $T_{\bar{p}}\approx 0.234{\langle p_{T}\rangle }_{\bar{p}}$, and $T_{p}\approx 0.240{\langle p_{T}\rangle }_{p}$. Here, the type of a particle appears as the subscript label of the related quantity. The ratio of $T/\langle p_{T}\rangle$ is approximately independent of a particle mass. This is consistent with the ratios of $T_{0}/\langle p_{T}\rangle$ and $T_{i}/\sqrt{\langle p_{T}^{2}\rangle }$, which are independent of particle mass according to Equations (17) and (18).

As only a free parameter, *T* does not show an obvious dependence on particle type or mass. However, it is hard to extract exact information from *T* because it is not a real temperature, because it also contains the contribution of transverse flow. $T_{0}$ is smaller than $T_{i}$ due to the fact that $T_{0}$ is “measured” at the kinetic freeze-out stage (the final one), and $T_{i}$ is “measured” at the initial stage. From the initial stage to the final one, the system becomes colder and colder. This is indeed observed in the present work.

In the above discussions, although chemical potential μ runs through the entire process, it is an insensitive quantity in the fit and not a free parameter due to the fact that it depends on $T_{ch}$ and $k_{j}$. Our previous work [52,53] shows that, from 6.3 to 17.3 GeV, $\mu_{\pi^{+}}$, $\mu_{K^{+}}$, and $\mu_{p}$ are around 0.041–0.017, 0.110–0.042, and 0.510–0.180 GeV, respectively, which are directly used in this work. These results have excluded the contributions from resonance decays [59]. Although the resonance decays contribute considerably to the yields of negative and positive hadrons, they contribute to the yield ratios, and then, the chemical potentials are small [52,53].

Before the summary and conclusions, it should be pointed out that the data sets analyzed by us are in a narrow and low-$p_{T}$ range and obey the standard distribution. We believe that even if the narrow spectra are in a high-$p_{T}$ range, the standard distribution can be used, and a high temperature can be obtained. The success of this work reflects that the classical concept and distribution can still play a great role in the field of high-energy collisions, though the application is in a local region. In our opinion, when researchers search for novel theoretical models, they first need to take into account classical theories.

Although the topic has been extensively studied in many papers for the SPS, RHIC, and LHC heavy-ion collisions and outline the validity of a nonextensive statistical distribution [60,61,62,63,64,65,66], those investigations used the spectra in a wide $p_{T}$ range. It is unanimous that for the wide $p_{T}$ spectra, a two-, three-, or multi-component standard distribution is needed in the fit. Then, a temperature fluctuation can be observed from the multi-component standard distribution. At this point, the Tsallis distribution is needed. This is the relationship between the standard distribution and the Tsallis distribution in the fit process.

In addition, in comparison with Hanbury–Brown–Twiss (HBT) results [67], large values of volume are obtained in the present work. The reason is that different volumes are studied. Generally, the former describes the system size in the initial state of collisions, and the latter is a reflection of the size of an expanded fireball in the final state (at the kinetic freeze-out) of collisions. Obviously, the latter is much larger than the former. The values of the three temperatures obtained in the present work seem reasonable.

## 4. Summary and Conclusions

The main observations and conclusions are summarized here.

(a) The transverse momentum spectra of the identified charged hadrons ($\pi^{-}$, $\pi^{+}$, $K^{-}$, $K^{+}$, $\bar{p}$, and *p*) with different rapidities produced in proton–proton collisions at center-of-mass energies $\sqrt{s}=6.3$, 7.7, 8.8, 12.3, and 17.3 GeV have been studied using the standard distribution. The fitted results are in agreement with the experimental data measured by the NA61/SHINE Collaboration at the SPS. The effective temperature *T*, kinetic freeze-out temperature $T_{0}$, initial temperature $T_{i}$, and kinetic freeze-out volume *V* are extracted. The present work shows that the standard distribution coming from the relativistic ideal gas model works well in some cases.

(b) In most cases, *T*, $T_{0}$, and $T_{i}$ decrease with the increase in rapidity *y* and increase with the increase in $\sqrt{s}$. There is a tendency of saturation for the three temperatures at $\sqrt{s}=7.7$ GeV and above. From a quick increase to a slow saturation in the three temperatures, the transition energy 7.7 GeV is the boundary for proton-dominated and meson-dominated final states. For the spectra of produced hadrons ($\pi^{-}$, $\pi^{+}$, $K^{-}$, $K^{+}$, and $\bar{p}$), the extracted *V* also decreases with the increase in *y* and increases with the increase in $\sqrt{s}$. For the spectra of *p*, the extracted *V* increases with the increase in *y* and decreases with the increase in $\sqrt{s}$. This is opposite to other hadrons because *p* contains the pre-existing leading protons, which affect the result.

(c) The three temperatures do not show an obvious isospin dependence. However, *V* shows a significant isospin dependence. The reason for the isospin dependence of *V* is the electromagnetic interactions between positive (negative) hadrons and pre-existing protons. The exclusion (attraction) between positive (negative) hadrons and pre-existing protons causes larger (smaller) *V* of an emission source of positive (negative) hadrons. Compared with the three temperature types, *V* shows a larger mass dependence. The mass dependence of *V* is also a reflection of a mass-dependent differential kinetic freeze-out scenario or multiple kinetic freeze-out scenario.

## Data Availability

The data used to support the findings of this study are included within the article and are cited at relevant places within the text as references.

## Appendix A. The Method to Obtain the Parameter and Its Uncertainty

Let

(A1) $$\begin{array}{c} y_{i}=f\left(x_{i}\right),\;i=1,2,\ldots ,n \end{array}$$

be the model value of the *i*-th fitting point. The physical quantities or parameters, $\lambda \;(\lambda_{1},\lambda_{2},\ldots ,\lambda_{j}$), can be obtained by fitting the experimental data, where *j* is the number of parameters, which includes the normalization constant. One has

(A2) $$\begin{array}{c} \chi^{2}=\sum_{i=1}^{n}\frac{{\left[f\left(x_{i}\right)-Y_{i}\right]}^{2}}{{\left(\delta Y_{i}\right)}^{2}}, \end{array}$$

where *n* is the number of fitting points, $Y_{i}$ represents the experimental value, and $\delta Y_{i}$ represents the uncertainty of the experimental value, usually including statistical and systematic uncertainties.

Due to the small particle number of $p_{T}$ samples being studied in this paper, the parameter uncertainty is assumed to follow the Student’s distribution (shortened to the t-distribution) [68],

(A3) $$\begin{array}{c} f\left(\lambda |\nu \right)=\frac{{\left(\frac{\nu }{\nu +\lambda^{2}}\right)}^{\frac{\nu +1}{2}}\Gamma \left(\frac{\nu +1}{2}\right)}{\sqrt{\mu \pi }\Gamma \left(\frac{\nu }{2}\right)}, \end{array}$$

where ν represents the ndof and Γ(x) represents the Gamma function. With the increase in ν, the t-distribution gradually approaches the normal or Gaussian distribution N(0,1). When ν approaches 1, the t-distribution approaches the Cauchy distribution.

In the present fitting, a 0.5% confidence interval is used to describe the parameter uncertainty. This means that there is a 0.5% probability that the parameter will fall within ($\lambda_{j}-t\sigma ,\lambda_{j}+t\sigma$), where σ is the standard deviation of each parameter and *t* satisfies the equation

(A4) $$\begin{array}{c} P=\int_{-t\sigma }^{t\sigma }f\left(\lambda \right)d\lambda =0.005. \end{array}$$

The standard deviation of each parameter can be calculated using

(A5) $$\begin{array}{c} \sigma =\sqrt{{\left(J_{\lambda }^{T}J_{\lambda }\right)}^{-1}\frac{s^{2}}{\nu }}, \end{array}$$

where

(A6) $$\begin{array}{c} {\left(J_{\lambda }\right)}_{ij}=\frac{\partial [f\left(x_{i}\right)-Y_{i}]}{\partial \lambda_{j}} \end{array}$$

is the Jacobian matrix and determinant of the model, $J_{\lambda }^{T}$ is the transpose of the Jacobian matrix, the superscript −1 represents matrix inversion, and

(A7) $$\begin{array}{c} s^{2}=\sum_{i=1}^{n}{\left[f\left(x_{i}\right)-Y_{i}\right]}^{2} \end{array}$$

is the variance. Then, the corresponding best parameter is given by

(A8) $$\begin{array}{c} \lambda_{j}\in \left[\lambda_{j}-t\sigma_{j,j},\lambda_{j}+t\sigma_{j,j}\right] \end{array}$$

with an uncertainty of $t\sigma_{j,j}$.

## Appendix B. Parameter Tables Obtained in the Fitting Process

**Table A1:** Values of *T*, *V*, $\chi^{2}$, and ndof corresponding to the curves in Figure 1 and Figure 2 for $\pi^{-}$ and $\pi^{+}$ produced in pp collisions, respectively, where the values of $\sqrt{s}$ are given together. The values of $\chi^{2}$ are reserved as an integer or the first non-zero decimal (if less than 1). The symbol “−” indicates that the data are not available.

$\sqrt{s}$ (GeV)	*y*	$\pi^{-}$			$\pi^{+}$		
		*T* (GeV)	*V* (${\mathrm{fm}}^{3}$)	$\chi^{2}$/ndof	*T* (GeV)	*V* (${\mathrm{fm}}^{3}$)	$\chi^{2}$/ndof
	0.1	0.118±0.001	$\left(8.119\pm 0.177\right)\times 10^{1}$	13/16	−	−	−
	0.3	0.123±0.001	$\left(7.755\pm 0.167\right)\times 10^{1}$	17/16	0.115±0.002	$\left(1.268\pm 0.072\right)\times 10^{2}$	0.09/6
	0.5	0.119±0.001	$\left(7.247\pm 0.157\right)\times 10^{1}$	15/16	0.108±0.001	$\left(9.805\pm 0.269\right)\times 10^{1}$	2/10
	0.7	0.118±0.001	$\left(6.514\pm 0.141\right)\times 10^{1}$	11/16	0.107±0.001	$\left(1.017\pm 0.028\right)\times 10^{2}$	1/10
	0.9	0.114±0.001	$\left(5.516\pm 0.121\right)\times 10^{1}$	11/16	0.098±0.001	$\left(1.007\pm 0.029\right)\times 10^{2}$	3/10
6.3	1.1	0.108±0.001	$\left(4.464\pm 0.098\right)\times 10^{1}$	16/16	0.100±0.001	$\left(7.106\pm 0.179\right)\times 10^{1}$	2/9
	1.3	0.097±0.001	$\left(3.466\pm 0.081\right)\times 10^{1}$	14/15	0.087±0.001	$\left(8.072\pm 0.468\right)\times 10^{1}$	1/6
	1.5	0.088±0.001	$\left(2.472\pm 0.061\right)\times 10^{1}$	9/14	0.088±0.001	$\left(5.903\pm 0.423\right)\times 10^{1}$	1/4
	1.7	−	−	−	0.081±0.001	$\left(3.957\pm 0.196\right)\times 10^{1}$	4/5
	1.9	−	−	−	0.070±0.001	$\left(2.736\pm 0.112\right)\times 10^{1}$	4/6
	2.1	−	−	−	0.066±0.001	$\left(2.036\pm 0.099\right)\times 10^{1}$	0.1/5
	0.1	0.123±0.001	$\left(9.450\pm 0.204\right)\times 10^{1}$	43/16	0.130±0.003	$\left(1.261\pm 0.137\right)\times 10^{2}$	0.5/3
	0.3	0.124±0.001	$\left(9.141\pm 0.197\right)\times 10^{1}$	33/16	0.121±0.001	$\left(1.423\pm 0.052\right)\times 10^{2}$	0.5/6
	0.5	0.122±0.001	$\left(8.475\pm 0.183\right)\times 10^{1}$	32/16	0.121±0.001	$\left(1.356\pm 0.037\right)\times 10^{2}$	2/9
	0.7	0.122±0.001	$\left(7.944\pm 0.172\right)\times 10^{1}$	19/16	0.119±0.001	$\left(1.253\pm 0.035\right)\times 10^{2}$	1/9
	0.9	0.119±0.001	$\left(6.865\pm 0.149\right)\times 10^{1}$	20/16	0.116±0.001	$\left(1.165\pm 0.032\right)\times 10^{2}$	2/9
7.7	1.1	0.115±0.001	$\left(5.715\pm 0.124\right)\times 10^{1}$	17/16	0.116±0.001	$\left(9.373\pm 0.251\right)\times 10^{1}$	0.5/8
	1.3	0.107±0.001	$\left(4.585\pm 0.104\right)\times 10^{1}$	14/15	0.108±0.001	$\left(8.131\pm 0.354\right)\times 10^{1}$	1/7
	1.5	0.101±0.001	$\left(3.526\pm 0.081\right)\times 10^{1}$	12/15	0.098±0.001	$\left(7.273\pm 0.343\right)\times 10^{1}$	1/7
	1.7	0.092±0.001	$\left(2.600\pm 0.066\right)\times 10^{1}$	12/13	0.086±0.001	$\left(5.933\pm 0.223\right)\times 10^{1}$	4/8
	1.9	−	−	−	0.075±0.001	$\left(3.963\pm 0.141\right)\times 10^{1}$	12/8
	2.1	−	−	−	0.069±0.001	$\left(2.440\pm 0.115\right)\times 10^{1}$	35/9
	0.1	0.124±0.001	$\left(1.015\pm 0.025\right)\times 10^{2}$	53/16	0.136±0.003	$\left(1.130\pm 0.104\right)\times 10^{2}$	0.2/4
	0.3	0.125±0.001	$\left(9.894\pm 0.219\right)\times 10^{1}$	70/16	0.124±0.001	$\left(1.460\pm 0.057\right)\times 10^{2}$	1/7
	0.5	0.124±0.001	$\left(9.394\pm 0.208\right)\times 10^{1}$	69/16	0.121±0.001	$\left(1.405\pm 0.044\right)\times 10^{2}$	1/8
	0.7	0.122±0.001	$\left(8.704\pm 0.193\right)\times 10^{1}$	43/16	0.123±0.001	$\left(1.318\pm 0.036\right)\times 10^{2}$	1/9
	0.9	0.126±0.001	$\left(7.596\pm 0.245\right)\times 10^{1}$	2/7	0.121±0.001	$\left(1.235\pm 0.035\right)\times 10^{2}$	1/9
8.8	1.1	0.119±0.001	$\left(6.808\pm 0.245\right)\times 10^{1}$	0.2/6	0.118±0.001	$\left(1.093\pm 0.031\right)\times 10^{2}$	0.4/9
	1.3	0.114±0.001	$\left(5.736\pm 0.229\right)\times 10^{1}$	1/5	0.113±0.001	$\left(9.364\pm 0.273\right)\times 10^{1}$	1/9
	1.5	0.102±0.001	$\left(4.687\pm 0.238\right)\times 10^{1}$	0.2/4	0.107±0.001	$\left(8.245\pm 0.270\right)\times 10^{1}$	0.2/8
	1.7	0.096±0.001	$\left(3.175\pm 0.076\right)\times 10^{1}$	11/15	0.096±0.001	$\left(6.446\pm 0.198\right)\times 10^{1}$	4/9
	1.9	0.086±0.001	$\left(2.337\pm 0.062\right)\times 10^{1}$	8/13	0.084±0.001	$\left(4.803\pm 0.152\right)\times 10^{1}$	4/9
	2.1	−	−	−	0.076±0.001	$\left(3.612\pm 0.137\right)\times 10^{1}$	3/7
	0.1	0.126±0.001	$\left(1.172\pm 0.025\right)\times 10^{2}$	88/16	0.132±0.002	$\left(1.351\pm 0.106\right)\times 10^{2}$	0.3/4
	0.3	0.126±0.001	$\left(1.149\pm 0.025\right)\times 10^{2}$	91/16	0.128±0.002	$\left(1.507\pm 0.076\right)\times 10^{2}$	0.4/6
	0.5	0.125±0.001	$\left(1.123\pm 0.024\right)\times 10^{2}$	69/16	0.131±0.001	$\left(1.379\pm 0.046\right)\times 10^{2}$	2/8
	0.7	0.124±0.001	$\left(1.034\pm 0.022\right)\times 10^{2}$	60/16	0.124±0.001	$\left(1.426\pm 0.046\right)\times 10^{2}$	0.4/7
	0.9	0.122±0.001	$\left(9.590\pm 0.206\right)\times 10^{1}$	51/16	0.121±0.001	$\left(1.334\pm 0.039\right)\times 10^{2}$	1/8
12.3	1.1	0.122±0.001	$\left(8.621\pm 0.185\right)\times 10^{1}$	43/16	0.122±0.001	$\left(1.208\pm 0.033\right)\times 10^{2}$	1/9
	1.3	0.118±0.001	$\left(7.327\pm 0.158\right)\times 10^{1}$	27/16	0.120±0.001	$\left(1.060\pm 0.026\right)\times 10^{2}$	1/11
	1.5	0.114±0.001	$\left(6.270\pm 0.136\right)\times 10^{1}$	19/16	0.118±0.001	$\left(9.376\pm 0.233\right)\times 10^{1}$	1/11
	1.7	0.109±0.001	$\left(5.063\pm 0.112\right)\times 10^{1}$	20/16	0.113±0.001	$\left(7.718\pm 0.020\right)\times 10^{1}$	2/11
	1.9	−	−	−	0.106±0.001	$\left(6.229\pm 0.016\right)\times 10^{1}$	3/11
	2.1	−	−	−	0.097±0.001	$\left(4.876\pm 0.014\right)\times 10^{1}$	4/10
	0.1	0.127±0.001	$\left(1.312\pm 0.028\right)\times 10^{2}$	89/16	0.137±0.003	$\left(1.234\pm 0.145\right)\times 10^{2}$	0.05/3
	0.3	0.129±0.001	$\left(1.293\pm 0.027\right)\times 10^{2}$	99/16	0.127±0.002	$\left(1.514\pm 0.127\right)\times 10^{2}$	0.1/4
	0.5	0.127±0.001	$\left(1.248\pm 0.026\right)\times 10^{2}$	93/16	0.128±0.002	$\left(1.469\pm 0.087\right)\times 10^{2}$	0.4/5
	0.7	0.127±0.001	$\left(1.176\pm 0.025\right)\times 10^{2}$	92/16	0.126±0.002	$\left(1.502\pm 0.064\right)\times 10^{2}$	1/6
	0.9	0.126±0.001	$\left(1.104\pm 0.023\right)\times 10^{2}$	87/16	0.125±0.002	$\left(1.442\pm 0.061\right)\times 10^{2}$	2/6
17.3	1.1	0.124±0.001	$\left(1.022\pm 0.021\right)\times 10^{2}$	66/16	0.125±0.001	$\left(1.342\pm 0.045\right)\times 10^{2}$	1/7
	1.3	0.122±0.001	$\left(9.378\pm 0.971\right)\times 10^{1}$	37/16	0.120±0.001	$\left(1.358\pm 0.045\right)\times 10^{2}$	1/7
	1.5	0.120±0.001	$\left(8.359\pm 0.177\right)\times 10^{1}$	27/16	0.120±0.001	$\left(1.189\pm 0.039\right)\times 10^{2}$	0.2/7
	1.7	0.116±0.001	$\left(7.247\pm 0.154\right)\times 10^{1}$	20/16	0.117±0.001	$\left(1.079\pm 0.035\right)\times 10^{2}$	0.4/7
	1.9	0.112±0.001	$\left(5.898\pm 0.126\right)\times 10^{1}$	16/16	0.120±0.001	$\left(9.162\pm 0.030\right)\times 10^{1}$	0.1/7
	2.1	0.106±0.001	$\left(4.619\pm 0.100\right)\times 10^{1}$	20/16	0.118±0.001	$\left(7.601\pm 0.024\right)\times 10^{1}$	0.2/7

**Table A2:** Values of *T*, *V*, $\chi^{2}$, and ndof corresponding to the curves in Figure 3 and Figure 4 for $K^{-}$ and $K^{+}$ produced in pp collisions, respectively.

$\sqrt{s}$ (GeV)	*y*	$K^{-}$			$K^{+}$		
		*T* (GeV)	*V* (${\mathrm{fm}}^{3}$)	$\chi^{2}$/ndof	*T* (GeV)	*V* (${\mathrm{fm}}^{3}$)	$\chi^{2}$/ndof
	0.1	0.121±0.001	$\left(3.013\pm 0.186\right)\times 10^{0}$	2/8	0.110±0.002	$\left(9.184\pm 0.990\right)\times 10^{0}$	1/5
	0.3	0.116±0.001	$\left(2.824\pm 0.195\right)\times 10^{0}$	1/7	0.108±0.002	$\left(9.001\pm 0.863\right)\times 10^{0}$	3/6
	0.5	0.115±0.002	$\left(2.422\pm 0.284\right)\times 10^{0}$	2/4	0.098±0.003	$\left(6.165\pm 1.215\right)\times 10^{0}$	6/4
6.3	0.7	0.081±0.001	$\left(1.748\pm 0.247\right)\times 10^{0}$	3/4	0.100±0.001	$\left(5.302\pm 0.461\right)\times 10^{0}$	18/10
	0.9	0.094±0.001	$\left(1.190\pm 0.108\right)\times 10^{0}$	4/6	0.091±0.001	$\left(5.201\pm 0.359\right)\times 10^{0}$	5/10
	1.1	0.098±0.002	$\left(5.859\pm 0.722\right)\times 10^{-1}$	4/4	0.099±0.001	$\left(2.675\pm 0.166\right)\times 10^{0}$	10/9
	1.3	0.096±0.005	$\left(4.229\pm 1.372\right)\times 10^{-1}$	0.09/2	0.081±0.001	$\left(2.467\pm 0.243\right)\times 10^{0}$	3/6
	1.5	0.073±0.003	$\left(2.041\pm 0.654\right)\times 10^{-1}$	0.7/2	0.078±0.001	$\left(1.935\pm 0.200\right)\times 10^{0}$	5/6
	0.1	0.130±0.002	$\left(4.775\pm 0.457\right)\times 10^{0}$	0.5/5	0.137±0.002	$\left(1.240\pm 0.087\right)\times 10^{1}$	1/7
	0.3	0.115±0.002	$\left(4.123\pm 0.335\right)\times 10^{0}$	2/6	0.138±0.002	$\left(1.211\pm 0.065\right)\times 10^{1}$	1/9
	0.5	0.136±0.003	$\left(3.434\pm 0.386\right)\times 10^{0}$	1/4	0.142±0.001	$\left(1.083\pm 0.084\right)\times 10^{1}$	1/6
	0.7	0.111±0.002	$\left(3.004\pm 0.244\right)\times 10^{0}$	1/6	0.146±0.001	$\left(9.481\pm 0.497\right)\times 10^{0}$	1/9
	0.9	0.120±0.002	$\left(2.526\pm 0.197\right)\times 10^{0}$	1/6	0.136±0.001	$\left(8.165\pm 0.439\right)\times 10^{0}$	1/9
7.7	1.1	0.116±0.002	$\left(1.703\pm 0.162\right)\times 10^{0}$	1/5	0.129±0.001	$\left(7.598\pm 0.423\right)\times 10^{0}$	1/9
	1.3	0.108±0.002	$\left(1.097\pm 0.108\right)\times 10^{0}$	1/5	0.130±0.001	$\left(6.466\pm 0.358\right)\times 10^{0}$	4/9
	1.5	0.099±0.003	$\left(6.563\pm 1.121\right)\times 10^{-1}$	0.3/3	0.120±0.001	$\left(5.394\pm 0.317\right)\times 10^{0}$	2/9
	1.7	0.063±0.002	$\left(2.875\pm 0.807\right)\times 10^{-1}$	0.4/2	0.110±0.001	$\left(4.168\pm 0.277\right)\times 10^{0}$	1/8
	1.9	−	−	−	0.095±0.001	$\left(3.140\pm 0.256\right)\times 10^{0}$	2/7
	2.1	−	−	−	0.078±0.001	$\left(2.172\pm 0.229\right)\times 10^{0}$	7/6
	0.1	0.124±0.002	$\left(5.460\pm 0.357\right)\times 10^{0}$	3/7	0.124±0.001	$\left(1.278\pm 0.072\right)\times 10^{1}$	3/9
	0.3	0.128±0.002	$\left(5.890\pm 0.431\right)\times 10^{0}$	0.4/6	0.126±0.001	$\left(1.259\pm 0.076\right)\times 10^{1}$	1/8
	0.5	0.133±0.002	$\left(4.015\pm 0.250\right)\times 10^{0}$	3/7	0.142±0.001	$\left(9.172\pm 0.468\right)\times 10^{0}$	2/9
	0.7	0.119±0.002	$\left(3.591\pm 0.245\right)\times 10^{0}$	1/7	0.139±0.001	$\left(8.748\pm 0.463\right)\times 10^{0}$	1/9
8.8	0.9	0.124±0.002	$\left(3.099\pm 0.239\right)\times 10^{0}$	0.4/6	0.140±0.001	$\left(8.885\pm 0.470\right)\times 10^{0}$	0.5/9
	1.1	0.134±0.002	$\left(2.426\pm 0.185\right)\times 10^{0}$	1/6	0.146±0.002	$\left(8.079\pm 0.418\right)\times 10^{0}$	1/9
	1.3	0.106±0.002	$\left(1.478\pm 0.144\right)\times 10^{0}$	1/5	0.146±0.001	$\left(6.816\pm 0.352\right)\times 10^{0}$	1/9
	1.5	0.089±0.002	$\left(9.181\pm 1.199\right)\times 10^{-1}$	0.2/4	0.131±0.001	$\left(5.690\pm 0.313\right)\times 10^{0}$	1/9
	1.7	0.091±0.002	$\left(5.613\pm 0.733\right)\times 10^{-1}$	2/4	0.128±0.001	$\left(4.577\pm 0.252\right)\times 10^{0}$	2/9
	1.9	0.080±0.003	$\left(3.513\pm 0.860\right)\times 10^{-1}$	1/2	0.132±0.001	$\left(3.929\pm 0.228\right)\times 10^{0}$	5/8
	0.1	0.125±0.002	$\left(7.420\pm 0.487\right)\times 10^{0}$	1/7	0.129±0.002	$\left(1.404\pm 0.093\right)\times 10^{1}$	1/7
	0.3	0.124±0.002	$\left(7.399\pm 0.563\right)\times 10^{0}$	0.3/6	0.139±0.002	$\left(1.419\pm 0.075\right)\times 10^{1}$	2/9
	0.5	0.132±0.002	$\left(6.808\pm 0.504\right)\times 10^{0}$	0.4/6	0.139±0.001	$\left(1.398\pm 0.073\right)\times 10^{1}$	1/9
	0.7	0.132±0.002	$\left(6.064\pm 0.452\right)\times 10^{0}$	0.4/6	0.152±0.002	$\left(1.278\pm 0.070\right)\times 10^{1}$	0.1/8
12.3	0.9	0.115±0.001	$\left(4.823\pm 0.380\right)\times 10^{0}$	0.5/6	0.139±0.002	$\left(1.128\pm 0.072\right)\times 10^{1}$	1/7
	1.1	0.113±0.001	$\left(3.704\pm 0.262\right)\times 10^{0}$	1/7	0.140±0.002	$\left(1.005\pm 0.523\right)\times 10^{1}$	0.4/9
	1.3	0.111±0.001	$\left(2.861\pm 0.207\right)\times 10^{0}$	0.4/7	0.137±0.001	$\left(8.783\pm 0.463\right)\times 10^{0}$	1/9
	1.5	0.113±0.002	$\left(2.064\pm 0.196\right)\times 10^{0}$	0.08/5	0.134±0.001	$\left(7.117\pm 0.375\right)\times 10^{0}$	0.4/9
	1.7	0.094±0.002	$\left(1.293\pm 0.169\right)\times 10^{0}$	2/4	0.133±0.001	$\left(6.053\pm 0.354\right)\times 10^{0}$	0.3/8
	0.1	0.137±0.002	$\left(9.281\pm 0.516\right)\times 10^{0}$	0.4/8	0.128±0.001	$\left(1.480\pm 0.088\right)\times 10^{1}$	2/8
	0.3	0.137±0.002	$\left(9.562\pm 0.601\right)\times 10^{0}$	0.05/7	0.129±0.002	$\left(1.512\pm 0.090\right)\times 10^{1}$	1/8
	0.5	0.130±0.002	$\left(8.807\pm 0.511\right)\times 10^{0}$	0.3/8	0.133±0.002	$\left(1.566\pm 0.092\right)\times 10^{1}$	3/8
	0.7	0.135±0.002	$\left(8.375\pm 0.527\right)\times 10^{0}$	0.1/7	0.149±0.002	$\left(1.543\pm 0.086\right)\times 10^{1}$	1/8
	0.9	0.136±0.001	$\left(7.181\pm 0.381\right)\times 10^{0}$	1/9	0.143±0.002	$\left(1.331\pm 0.075\right)\times 10^{1}$	0.2/8
17.3	1.1	0.121±0.001	$\left(6.036\pm 0.346\right)\times 10^{0}$	1/44	0.139±0.002	$\left(1.149\pm 0.065\right)\times 10^{1}$	0.4/8
	1.3	0.115±0.001	$\left(4.485\pm 0.283\right)\times 10^{0}$	2/8	0.140±0.002	$\left(9.986\pm 0.633\right)\times 10^{0}$	0.1/7
	1.5	0.115±0.001	$\left(3.557\pm 0.225\right)\times 10^{0}$	0.5/8	0.133±0.002	$\left(8.618\pm 0.554\right)\times 10^{0}$	1/7
	1.7	0.102±0.001	$\left(2.318\pm 0.189\right)\times 10^{0}$	4/6	0.127±0.002	$\left(6.873\pm 0.449\right)\times 10^{0}$	0.4/7
	1.9	0.098±0.002	$\left(1.667\pm 0.167\right)\times 10^{0}$	0.1/5	0.124±0.002	$\left(5.261\pm 0.401\right)\times 10^{0}$	1/6
	2.1	0.076±0.001	$\left(0.833\pm 0.115\right)\times 10^{0}$	2/4	0.118±0.002	$\left(2.968\pm 0.205\right)\times 10^{0}$	6/7

**Table A3:** Values of *T*, *V*, $\chi^{2}$, and ndof corresponding to the curves in Figure 5 and Figure 6 for $\bar{p}$ and *p* produced in pp collisions, respectively.

$\sqrt{s}$ (GeV)	*y*	$\bar{p}$			*p*		
		*T* (GeV)	*V* (${\mathrm{fm}}^{3}$)	$\chi^{2}$/ndof	*T* (GeV)	*V* (${\mathrm{fm}}^{3}$)	$\chi^{2}$/ndof
	0.1	−	−	−	0.104±0.001	$\left(1.662\pm 0.245\right)\times 10^{1}$	4/7
	0.3	−	−	−	0.095±0.001	$\left(1.993\pm 0.244\right)\times 10^{1}$	9/10
	0.5	−	−	−	0.091±0.001	$\left(2.176\pm 0.286\right)\times 10^{1}$	8/10
	0.7	−	−	−	0.097±0.001	$\left(2.359\pm 0.273\right)\times 10^{1}$	17/10
6.3	0.9	−	−	−	0.089±0.001	$\left(3.633\pm 0.520\right)\times 10^{1}$	4/9
	1.1	−	−	−	0.090±0.001	$\left(3.326\pm 0.630\right)\times 10^{1}$	3/6
	1.3	−	−	−	0.074±0.001	$\left(4.321\pm 0.963\right)\times 10^{1}$	1/6
	1.5	−	−	−	0.064±0.001	$\left(4.180\pm 1.066\right)\times 10^{1}$	2/6
	1.7	−	−	−	0.043±0.001	$\left(2.855\pm 1.225\right)\times 10^{1}$	0.3/6
	0.1	0.114±0.002	$\left(3.326\pm 0.458\right)\times 10^{-1}$	9/7	0.135±0.002	$\left(1.270\pm 0.121\right)\times 10^{1}$	1/9
	0.3	0.109±0.002	$\left(3.236\pm 0.457\right)\times 10^{-1}$	14/7	0.133±0.002	$\left(1.405\pm 0.136\right)\times 10^{1}$	1/9
	0.5	0.109±0.002	$\left(2.025\pm 0.332\right)\times 10^{-1}$	10/6	0.133±0.002	$\left(1.704\pm 0.165\right)\times 10^{1}$	1/9
	0.7	0.111±0.003	$\left(1.433\pm 0.301\right)\times 10^{-1}$	10/5	0.125±0.002	$\left(2.061\pm 0.211\right)\times 10^{1}$	0.3/9
7.7	0.9	0.065±0.001	$\left(6.943\pm 1.945\right)\times 10^{-2}$	12/5	0.124±0.002	$\left(2.637\pm 0.272\right)\times 10^{1}$	0.2/9
	1.1	−	−	−	0.126±0.002	$\left(3.075\pm 0.302\right)\times 10^{1}$	2/9
	1.3	−	−	−	0.114±0.001	$\left(3.436\pm 0.384\right)\times 10^{1}$	1/9
	1.5	−	−	−	0.104±0.001	$\left(3.801\pm 0.505\right)\times 10^{1}$	1/8
	1.7	−	−	−	0.086±0.001	$\left(4.103\pm 0.783\right)\times 10^{1}$	1/6
	0.1	0.105±0.003	$\left(3.835\pm 1.138\right)\times 10^{-1}$	6/3	0.135±0.002	$\left(1.318\pm 0.125\right)\times 10^{1}$	0.2/9
	0.3	0.112±0.002	$\left(3.713\pm 0.535\right)\times 10^{-1}$	4/7	0.137±0.002	$\left(1.417\pm 0.134\right)\times 10^{1}$	0.3/9
	0.5	0.104±0.002	$\left(2.982\pm 0.647\right)\times 10^{-1}$	4/5	0.133±0.002	$\left(1.632\pm 0.156\right)\times 10^{1}$	1/9
	0.7	0.131±0.002	$\left(1.682\pm 0.241\right)\times 10^{-1}$	29/6	0.127±0.002	$\left(1.928\pm 0.194\right)\times 10^{1}$	0.1/9
8.8	0.9	0.094±0.002	$\left(8.812\pm 1.583\right)\times 10^{-2}$	11/6	0.128±0.002	$\left(2.374\pm 0.236\right)\times 10^{1}$	0.08/9
	1.1	−	−	−	0.127±0.002	$\left(2.931\pm 0.293\right)\times 10^{1}$	0.08/9
	1.3	−	−	−	0.125±0.002	$\left(3.365\pm 0.342\right)\times 10^{1}$	1/9
	1.5	−	−	−	0.112±0.001	$\left(3.630\pm 0.411\right)\times 10^{1}$	0.2/9
	1.7	−	−	−	0.097±0.001	$\left(3.748\pm 0.584\right)\times 10^{1}$	1/7
	1.9	−	−	−	0.085±0.001	$\left(3.785\pm 0.730\right)\times 10^{1}$	1/6
	0.1	0.100±0.001	$\left(9.128\pm 1.170\right)\times 10^{-1}$	11/9	0.145±0.002	$\left(7.934\pm 0.667\right)\times 10^{0}$	1/10
	0.3	0.101±0.001	$\left(9.005\pm 1.106\right)\times 10^{-1}$	5/9	0.148±0.002	$\left(8.276\pm 0.678\right)\times 10^{0}$	1/10
	0.5	0.104±0.002	$\left(7.481\pm 0.905\right)\times 10^{-1}$	2/9	0.141±0.002	$\left(9.202\pm 0.872\right)\times 10^{0}$	1/9
	0.7	0.112±0.001	$\left(6.565\pm 0.747\right)\times 10^{-1}$	2/9	0.145±0.002	$\left(1.100\pm 0.101\right)\times 10^{1}$	0.2/9
12.3	0.9	0.103±0.001	$\left(4.409\pm 0.586\right)\times 10^{-1}$	5/8	0.142±0.002	$\left(1.324\pm 0.105\right)\times 10^{1}$	1/11
	1.1	0.111±0.001	$\left(2.464\pm 0.303\right)\times 10^{-1}$	4/8	0.135±0.001	$\left(1.683\pm 0.139\right)\times 10^{1}$	0.1/11
	1.3	0.103±0.002	$\left(1.558\pm 0.232\right)\times 10^{-1}$	8/7	0.132±0.001	$\left(2.091\pm 0.177\right)\times 10^{1}$	0.3/11
	1.5	0.100±0.002	$\left(7.733\pm 1.344\right)\times 10^{-2}$	11/6	0.127±0.001	$\left(2.468\pm 0.218\right)\times 10^{1}$	1/11
	1.7	−	−	−	0.119±0.001	$\left(2.891\pm 0.274\right)\times 10^{1}$	2/11
	1.9	−	−	−	0.104±0.001	$\left(3.098\pm 0.434\right)\times 10^{1}$	1/8
	0.1	0.126±0.002	$\left(1.719\pm 0.206\right)\times 10^{0}$	0.4/8	0.150±0.002	$\left(7.374\pm 0.755\right)\times 10^{0}$	0.2/8
	0.3	0.102±0.001	$\left(1.499\pm 0.174\right)\times 10^{0}$	11/10	0.150±0.002	$\left(7.430\pm 0.760\right)\times 10^{0}$	0.2/8
	0.5	0.126±0.001	$\left(1.544\pm 0.149\right)\times 10^{0}$	3/10	0.150±0.002	$\left(7.882\pm 0.810\right)\times 10^{0}$	0.08/8
	0.7	0.133±0.002	$\left(1.438\pm 0.144\right)\times 10^{0}$	1/9	0.143±0.002	$\left(9.096\pm 0.964\right)\times 10^{0}$	0.2/8
	0.9	0.121±0.002	$\left(1.086\pm 0.136\right)\times 10^{0}$	2/8	0.151±0.002	$\left(1.061\pm 0.109\right)\times 10^{1}$	0.5/8
17.3	1.1	0.127±0.002	$\left(0.805\pm 0.111\right)\times 10^{0}$	1/7	0.141±0.002	$\left(1.191\pm 0.149\right)\times 10^{1}$	0.09/7
	1.3	0.123±0.002	$\left(0.568\pm 0.066\right)\times 10^{0}$	4/8	0.138±0.002	$\left(1.555\pm 0.196\right)\times 10^{1}$	0.04/7
	1.5	0.161±0.005	$\left(0.410\pm 0.081\right)\times 10^{0}$	1/5	0.135±0.002	$\left(1.950\pm 0.248\right)\times 10^{1}$	0.02/7
	1.7	0.136±0.005	$\left(0.214\pm 0.064\right)\times 10^{0}$	2/4	0.136±0.002	$\left(2.515\pm 0.392\right)\times 10^{1}$	0.2/6
	1.9	0.071±0.003	$\left(0.057\pm 0.029\right)\times 10^{0}$	1/3	0.142±0.003	$\left(2.868\pm 0.491\right)\times 10^{1}$	0.001/5
	2.1	−	−	−	0.132±0.003	$\left(3.033\pm 0.531\right)\times 10^{1}$	0.4/5

## References

[1] Arsene I., Bearden I.G., Beavis D., Besliu C., Budick B., Bggild H., Chasman C., Chasman C., Christensen C.H., Christiansen P. (2005). Quark gluon plasma and color glass condensate at RHIC? The perspective from the BRAHMS experiment. Nucl. Phys. A.

[2] Adcox K., Adler S.S., Afanasiev S., Aidala C., Ajitanand N.N., Akiba Y., Al-Jamel A., Alexander J., Amirikas R., Aoki K. (2005). Formation of dense partonic matter in relativistic nucleus-nucleus collisions at RHIC: Experimental evaluation by the PHENIX collaboration. Nucl. Phys. A.

[3] Adams J., Aggarwal M.M., Ahammed Z., Amonett J., Anderson B.D., Arkhipkin D., Averichev G.S., Badyal S.K., Bai Y., Balewski J. (2005). Experimental and theoretical challenges in the search for the quark gluon plasma: The STAR collaboration’s critical assessment of the evidence from RHIC collisions. Nucl. Phys. A.

[4] Schukraft J. (2012). Heavy ion physics with the ALICE experiment at the CERN LHC. Phil. Trans. R. Soc. Lond. A.

[5] Braun-Munzinger P., Stachel J. (2007). The quest for the quark-gluon plasma. Nature.

[6] Harris J.W., Müller B. (1996). The search for the quark-gluon plasma. Ann. Rev. Nucl. Part. Sci..

[7] Heinz U.W., Jacob M. (2000). Evidence for a new state of matter: An assessment of the results from the CERN lead beam programme. arXiv.

[8] Podlaski P. (2023). Results on system size dependence of strangeness production in the CERN SPS energy range from NA61/SHINE. EPJ Web Conf..

[9] Tannenbaum M.J. (2006). Recent results in relativistic heavy ion collisions: From ‘a new state of matter’ to ‘the perfect fluid’. Rept. Prog. Phys..

[10] Abdulhamid M.I., Aboona B.E., Adam J., Adamczyk L., Adams J.R., Aggarwal I., Aggarwal M.M., Ahammed Z., Anderson D.M., Aschenauer E.C. (2023). Energy dependence of intermittency for charged hadrons in Au+Au collisions at RHIC. Phys. Lett. B.

[11] Abdulhamid M.I., Aboona B.E., Adam J., Adamczyk L., Adams J.R., Agakishiev G., Aggarwal I., Aggarwal M.M., Ahammed Z., Aitbaev A. (2023). Beam energy dependence of triton production and yield ratio (N_t_ × N_p_/$N_{d}^{2}$) in Au+Au collisions at RHIC. Phys. Rev. Lett..

[12] Aboona B.E., Adam J., Adamczyk L., Adams J.R., Aggarwal I., Aggarwal M.M., Ahammed Z., Anderson D.M., Aschenauer E.C., Atchison J. (2023). Search for the chiral magnetic effect in Au+Au collisions at $\sqrt{s_{NN}}$ = 27 GeV with the STAR forward event plane detectors. Phys. Lett. B.

[13] Adam J., Adamczyk L., Adams J.R., Adkins J.K., Agakishiev G., Aggarwal M.M., Ahammed Z., Alekseev I., Anderson D.M., Aoyama R. (2020). Bulk properties of the system formed in *Au* + *Au* collisions at $\sqrt{s_{NN}}$ = 14.5 GeV at the BNL STAR detector. Phys. Rev. C.

[14] Helmut S. (2003). Limits of confinement: The first 15 years of ultra-relativistic heavy ion studies. Nucl. Phys. A.

[15] Back B.B., Baker M.D., Ballintijn M., Barton D.S., Becker B., Betts R.R., Bickley A.A., Bindel R., Budzanowski A., Busza W. (2005). The PHOBOS perspective on discoveries at RHIC. Nucl. Phys. A.

[16] Aamodt K., Abelev B., Quintana A.A., Adamová D., Adare A.M., Aggarwal M.M., Rinella G.A., Agocs A.G., Salazar S.A., Ahammed Z. (2010). Elliptic flow of charged particles in Pb-Pb collisions at 2.76 TeV. Phys. Rev. Lett..

[17] Aamodt K., Abelev B., Quintana A.A., Adamová D., Adare A.M., Aggarwal M.M., Rinella G.A., Agocs A.G., Salazar S.A., Ahammed Z. (2010). Charged-particle multiplicity density at mid-rapidity in central Pb-Pb collisions at $\sqrt{s_{NN}}$ = 2.76 TeV. Phys. Rev. Lett..

[18] Aamodt K., Quintana A.A., Adamová D., Adare A.M., Aggarwal M.M., Rinella G.A., Agocs A.G., Salazar S.A., Ahammed Z., Ahmad N. (2011). Centrality dependence of the charged-particle multiplicity density at mid-rapidity in Pb-Pb collisions at $\sqrt{s_{NN}}$ = 2.76 TeV. Phys. Rev. Lett..

[19] Aamodt K., Abelev B., Quintana A.A., Adamová D., Adare A.M., Aggarwal M.M., Rinella G.A., Agocs A.G., Agostinelli A., Salazar S.A. (2011). Higher harmonic anisotropic flow measurements of charged particles in Pb-Pb collisions at $\sqrt{s_{NN}}$ = 2.76 TeV. Phys. Rev. Lett..

[20] Adler S.S., Afanasiev S., Aidala C., Ajitanand N.N., Akiba Y., Alexander J., Amirikas R., Aphecetche L., Aronson S.H., Averbeck R. (2004). Identified charged particle spectra and yields in Au+Au collisions at $\sqrt{s_{NN}}$ = 200 GeV. Phys. Rev. C.

[21] Van Hove L. (1982). Multiplicity dependence of *p_t_* spectrum as a possible signal for a phase transition in hadronic collisions. Phys. Lett. B.

[22] Andronic A., Braun-Munzinger P., Stachel J. (2006). Hadron production in central nucleus-nucleus collisions at chemical freeze-out. Nucl. Phys. A.

[23] Cleymans J., Oeschler H., Redlich K., Wheaton S. (2006). Comparison of chemical freeze-out criteria in heavy-ion collisions. Phys. Rev. C.

[24] Andronic A., Braun-Munzinger P., Stachel J. (2009). Thermal hadron production in relativistic nuclear collisions. Acta Phys. Pol. B.

[25] Andronic A., Braun-Munzinger P., Stachel J. (2010). The horn, the hadron mass spectrum and the QCD phase diagram: The statistical model of hadron production in central nucleus-nucleus collisions. Nucl. Phys. A.

[26] Redlich K., Cleymans J., Oeschler H., Tounsi A. (2002). Particle production and equilibration in heavy ion collisions. Acta Phys. Polon. B.

[27] Wheaton S., Cleymans J., Hauer M. (2009). THERMUS: A thermal model package for ROOT. Comput. Phys. Commun..

[28] Andronic A., Beutler F., Braun-Munzinger P., Redlich K., Stachel J. (2009). Statistical hadronization of heavy flavor quarks in elementary collisions: Successes and failures. Phys. Lett. B.

[29] Aduszkiewicz A., Ali Y., Andronov E., Antićić T., Baatar B., Baszczyk M., Bhosale S., Blondel A., Bogomilov M., Brandin A. (2017). Measurements of *π*^±^, *K*^±^, *p* and $\bar{p}$ spectra in proton–proton interactions at 20, 31, 40, 80 and 158 GeV/*c* with the NA61/SHINE spectrometer at the CERN SPS. Eur. Phys. J. C.

[30] Cleymans J., Worku D. (2012). Relativistic thermodynamics: Transverse momentum distributions in high-energy physics. Eur. Phys. J. A.

[31] Abelev B.I., Adams J., Aggarwal M.M., Ahammed Z., Amonett J., Anderson B.D., Anderson M., Arkhipkin D., Averichev G.S., Bai Y. (2007). Strange particle production in *p* + *p* collisions at $\sqrt{s}$ = 200 GeV. Phys. Rev. C.

[32] Tsallis C. (1988). Possible generalization of Boltzmann-Gibbs statistics. J. Stat. Phys..

[33] Biro T.S., Purcsel G., Urmossy K. (2009). Non-extensive approach to quark matter. Eur. Phys. J. A.

[34] Zheng H., Zhu L.L., Bonasera A. (2015). Systematic analysis of hadron spectra in *p* + *p* collisions using Tsallis distributions. Phys. Rev. D.

[35] Zheng H., Zhu L.L. (2015). Can Tsallis distribution fit all the particle spectra produced at RHIC and LHC?. Adv. High Energy Phys..

[36] Wang X.N., Hwa R.C. (1989). The effect of jet production on the multiplicity dependence of average transverse momentum. Phys. Rev. D.

[37] Sjöstrand T., van Zijl M. (1987). A multiple-interaction model for the event structure in hadron collisions. Phys. Rev. D.

[38] Parvan A.S. (2015). Non-extensive statistics effects in transverse momentum spectra of hadrons. arXiv.

[39] Rath R., Khuntia A., Sahoo R., Cleymans J. (2020). Event multiplicity, transverse momentum and energy dependence of charged particle production, and system thermodynamics in *pp* collisions at the Large Hadron Collider. J. Phys. G.

[40] Schnedermann E., Sollfrank J., Heinz U. (1993). Thermal phenomenology of hadrons from 200A GeV S+S collisions. Phys. Rev. C.

[41] Hagedorn R. (1983). Multiplicities, *p_T_* distributions and the expected hadron ⟶ quark-gluon phase transition. Riv. Nuovo Cimento.

[42] Adamczyk L., Adkins J.K., Agakishiev G., Aggarwal M.M., Ahammed Z., Ajitanand N.N., Alekseev I., Anderson D.M., Aoyama R., Aparin A. (2017). Bulk properties of the medium produced in relativistic heavy-ion collisions from the beam energy scan program. Phys. Rev. C.

[43] Braun-Munzinger P., Stachel J., Wessels J.P., Xu N. (1995). Thermal equilibration and expansion in nucleus-nucleus collisions at the AGS. Phys. Lett. B.

[44] Andronic A., Braun-Munzinger P., Stachel J. (2009). Thermal hadron production in relativistic nuclear collisions: The hadron mass spectrum, the horn, and the QCD phase transition. Phys. Lett. B.

[45] Abelev B.I., Aggarwal M.M., Ahammed Z., Anderson B.D., Arkhipkin D., Averichev G.S., Bai Y., Balewski J., Barannikova O., Barnby L.S. (2009). Systematic measurements of identified particle spectra in *pp*, *d*+Au and Au+Au collisions from STAR. Phys. Rev. C.

[46] Cleymans J., Oeschler H., Redlich K. (1999). Influence of impact parameter on thermal description of relativistic heavy ion collisions at (1–2)A GeV. Phys. Rev. C.

[47] Braun-Munzinger P., Heppe I., Stachel J. (1999). Chemical equilibration in Pb+Pb collisions at the SPS. Phys. Lett. B.

[48] Manninen J., Becattini F. (2008). Chemical freeze-out in ultra-relativistic heavy ion collisions at $\sqrt{s_{NN}}$ = 130 and 200 GeV. Phys. Rev. C.

[49] Andronic A., Braun-Munzinger P., Redlich K. (2018). Decoding the phase structure of QCD via particle production at high energy. Nature.

[50] Koch P., Rafelski J., Greiner W. (1983). Strange hadron in hot nuclear matter. Phys. Lett. B.

[51] Braun-Munzinger P., Magestro D., Redlich K., Stachel J. (2001). Hadron production in Au-Au collisions at RHIC. Phys. Lett. B.

[52] Lao H.-L., Gao Y.-Q., Liu F.-H. (2019). Energy dependent chemical potentials of light particles and quarks from yield ratios of antiparticles to particles in high energy collisions. Universe.

[53] Lao H.-L., Gao Y.-Q., Liu F.-H. (2020). Light particle and quark chemical potentials from negatively to positively charged particle yield ratios corrected by removing strong and weak decays. Adv. High Energy Phys..

[54] Gardim F.G., Giacalone G., Luzum M., Ollitrault J.Y. (2020). Thermodynamics of hot strong-interaction matter from ultrarelativistic nuclear collisions. Nat. Phys..

[55] Waqas M., Peng G.-X., Ajaz M., Haj Ismail A., Wazir Z., Li L.-L. (2022). Extraction of different temperatures and kinetic freeze-out volume in high energy collisions. J. Phys. G.

[56] Gutay L.J., Hirsch A.S., Pajares C., Scharenberg R.P., Srivastava B.K. (2015). De-confinement in small systems: Clustering of color sources in high multiplicity $\bar{p}$*p* collisions at $\sqrt{s}$ = 1.8 TeV. Int. J. Mod. Phys. E.

[57] Scharenberg R.P., Srivastava B.K., Pajares C. (2019). Exploring the initial stage of high multiplicity proton–proton collisions by determining the initial temperature of the quark-gluon plasma. Phys. Rev. D.

[58] Sahoo P., De S., Tiwari S.K., Sahoo R. (2018). Energy and centrality dependent study of deconfinement phase transition in a color string percolation approach at RHIC energies. Eur. Phys. J. A.

[59] Yu N., Luo X.F. (2019). Particle decay from statistical thermal model in high-energy nucleus-nucleus collisions. Eur. Phys. J. A.

[60] Biyajima M., Kaneyama M., Mizoguchi T., Wilk G. (2005). Analyses of *k_t_* distributions at RHIC by means of some selected statistical and stochastic models. Eur. Phys. J. C.

[61] Cleymans J., Hamar G., Levai P., Wheaton S. (2009). Near-thermal equilibrium with Tsallis distributions in heavy-ion collisions. J. Phys. G.

[62] Shao M., Yi L., Tang Z.B., Chen H.F., Li C., Xu Z.B. (2010). Examination of the species and beam energy dependence of particle spectra using Tsallis statistics. J. Phys. G.

[63] Wong C.-Y., Wilk G., Cirto L.J.L., Tsallis C. (2015). From QCD-based hard-scattering to nonextensive statistical mechanical descriptions of transverse momentum spectra in high-energy *pp* and *p*$\bar{p}$ collisions. Phys. Rev. D.

[64] Hui J.-Q., Jiang Z.-J., Xu D.-F. (2018). A description of the transverse momentum distributions of charged particles produced in heavy ion collisions at RHIC and LHC energies. Adv. High Energy Phys..

[65] Tripathy S., Tiwari S.K., Younus M., Sahoo R. (2018). Elliptic flow in Pb+Pb collisions at $\sqrt{s_{NN}}$ = 2.76 TeV at the LHC using Boltzmann transport equation with non-extensive statistics. Eur. Phys. J. A.

[66] Rybczyński M., Włodarczyk Z. (2014). Tsallis statistics approach to the transverse momentum distributions in p-p collisions. Eur. Phys. J. C.

[67] Zhang S., Ma Y.G., Chen J.H., Zhong C. (2016). Beam energy dependence of Hanbury-Brown-Twiss radii from a blast-wave model. Adv. High Energy Phys..

[68] Hogg R.V., McKean J.W., Craig A.T. (2018). Introduction to Mathematical Statistics.

